# Applying Spatial Metabolomics To Investigate Age- and Drug-Induced Neurochemical Changes

**DOI:** 10.1021/acschemneuro.4c00199

**Published:** 2024-07-29

**Authors:** Theodosia Vallianatou, Tina B. Angerer, Ibrahim Kaya, Anna Nilsson, Reza Shariatgorji, Per Svenningsson, Per E. Andrén

**Affiliations:** † Department of Pharmaceutical Biosciences, Spatial Mass Spectrometry, Science for Life Laboratory, Uppsala University, Uppsala SE-75124, Sweden; ‡ Department of Clinical Neuroscience, Karolinska Institute, Stockholm SE-17177, Sweden

**Keywords:** aging, acetylcholinesterase inhibitor, brain, lipids, mass spectrometry imaging, metabolites, sulfatides, tacrine

## Abstract

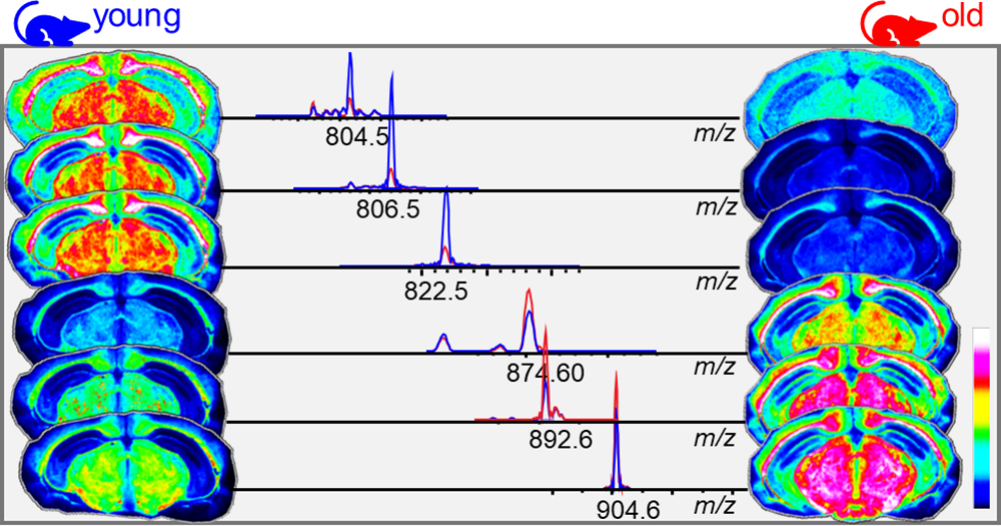

In an era when population aging is increasing the burden of neurodegenerative conditions, deciphering the mechanisms underlying brain senescence is more important than ever. Here, we present a spatial metabolomics analysis of age-induced neurochemical alterations in the mouse brain using negative ionization mode mass spectrometry imaging. The age-dependent effects of the acetylcholinesterase inhibitor tacrine were simultaneously examined. For ultrahigh mass resolution analysis, we utilized a Fourier-transform ion cyclotron resonance spectrometer. To complement this, a trapped ion mobility spectrometry time-of-flight analyzer provided high speed and lateral resolution. The chosen approach facilitated the detection and identification of a wide range of metabolites, from amino acids to sphingolipids. We reported significant, age-dependent alterations in brain lipids which were most evident for sulfatides and lysophosphatidic acids. Sulfatide species, which are mainly localized to white matter, either increased or decreased with age, depending on the carbon chain length and hydroxylation stage. Lysophosphatidic acids were found to decrease with age in the detailed cortical and hippocampal subregions. An age-dependent increase in the glutamine/glutamate ratio, an indicator of glia-neuron interconnection and neurotoxicity, was detected after tacrine administration. The presented metabolic mapping approach was able to provide visualizations of the lipid signaling and neurotransmission alterations induced by early aging and can thus be beneficial to further elucidating age-related neurochemical pathways.

## Introduction

Mass spectrometry imaging (MSI)-enabled spatial metabolomics has emerged as a powerful tool for the on-tissue mapping of thousands of metabolites, including lipids, metabolic intermediates, neurotransmitters, and peptides.^1−6^ The technique has demonstrated applications in several tissue types, with most of the research focus centering around brain and cancer tissues; this is because these tissues are characterized by anatomical and cellular heterogeneity which requires detailed mapping. Moreover, MSI-based spatial metabolomics has recently been combined with other tissue mapping modalities, such as spatial transcriptomics, for the simultaneous spatial profiling of small molecules and gene expression within a tissue section.^7^ Matrix-assisted laser desorption/ionization mass spectrometry imaging (MALDI-MSI) is the most widely used MSI technique. In addition, the ultrahigh mass resolution and mass accuracy provided by approaches such as Fourier-transform ion cyclotron resonance (FTICR) allow for untargeted spatial metabolomics. The recent development of high-frequency laser sources featuring structured beams can facilitate near-single-cell analysis of metabolites and lipids when combined with high scan rate mass analyzers^8,9^

Aging is a multifactorial process that is recognized as a major risk factor for the development of several neurodegenerative disorders, including Alzheimer’s (AD) and Parkinson’s disease (PD).^10^ Therefore, the metabolic profiling of normal brain aging can unveil the pathophysiological mechanisms underlying cellular senescence.^11−13^ Previous evidence has demonstrated that brain aging involves regional differences in multiple metabolic pathways that involve sphingolipids, neurotransmitters, and acylcarnitines.^12,14^ Although liquid chromatography–mass spectrometry-based metabolomics has traditionally been applied for detecting age-induced metabolic perturbations in the brain, MSI can provide detailed spatial information while retaining the sensitivity and selectivity of MS.

In previous studies, we used positive ionization mode MSI to identify various metabolic pathways that are associated with early brain aging via the detection and quantification of acylcarnitines, hexosyl-ceramides, carnosine, α-tocopherol, as well as cholinergic, dopaminergic, noradrenergic and histaminergic metabolites.^14−16^ Here, we focus on lipids, neurotransmitters, and metabolic intermediates that can be detected using negative ionization mode MALDI-MSI. We applied spatial metabolomics to reveal the potential involvement of these metabolites in early brain aging. Numerous brain lipids, such as sulfatides and lysophosphatidic acids, demonstrated significant, age-dependent alterations. In addition, the glutamine/glutamate ratio was also found to be affected by age.

## Results

### Detection and Identification of Brain Metabolites via MALDI-MSI

The applied MALDI-MSI method, run under negative ionization mode, enabled the detection and identification of a wide range of brain metabolites, ranging from hydrophilic amino acids to multiple classes of brain lipids (Figures S1 and S2). The small hydrophilic metabolites were identified by comparing the tandem MS (MS/MS) MALDI mass spectra collected from brain tissue samples with MS/MS spectra representing standards or available from experimentally derived databases.^17^ The identified metabolites represented a number of different metabolic pathways, such as neurotransmitters and metabolic intermediates, e.g., taurine, aspartate, glutamate, glutamine, *N*-acetyl aspartate, hypoxanthine, glutathione, and ascorbic acid (Figures S1 and S2). The reported identities of brain lipids were based on both high mass accuracy results and comparisons with the distributions of specific lipids provided in the prior literature.^18−20^ Selected sulfatides were identified using multiple reaction monitoring (MRM) desorption electrospray ionization (DESI)-MSI. The detection and identification of these molecules provided a useful overview and initial mapping of the mouse brain metabolome, which set the basis for subsequent metabolomics analysis.

### Spatial Brain Metabolomics

**Figure 1:**
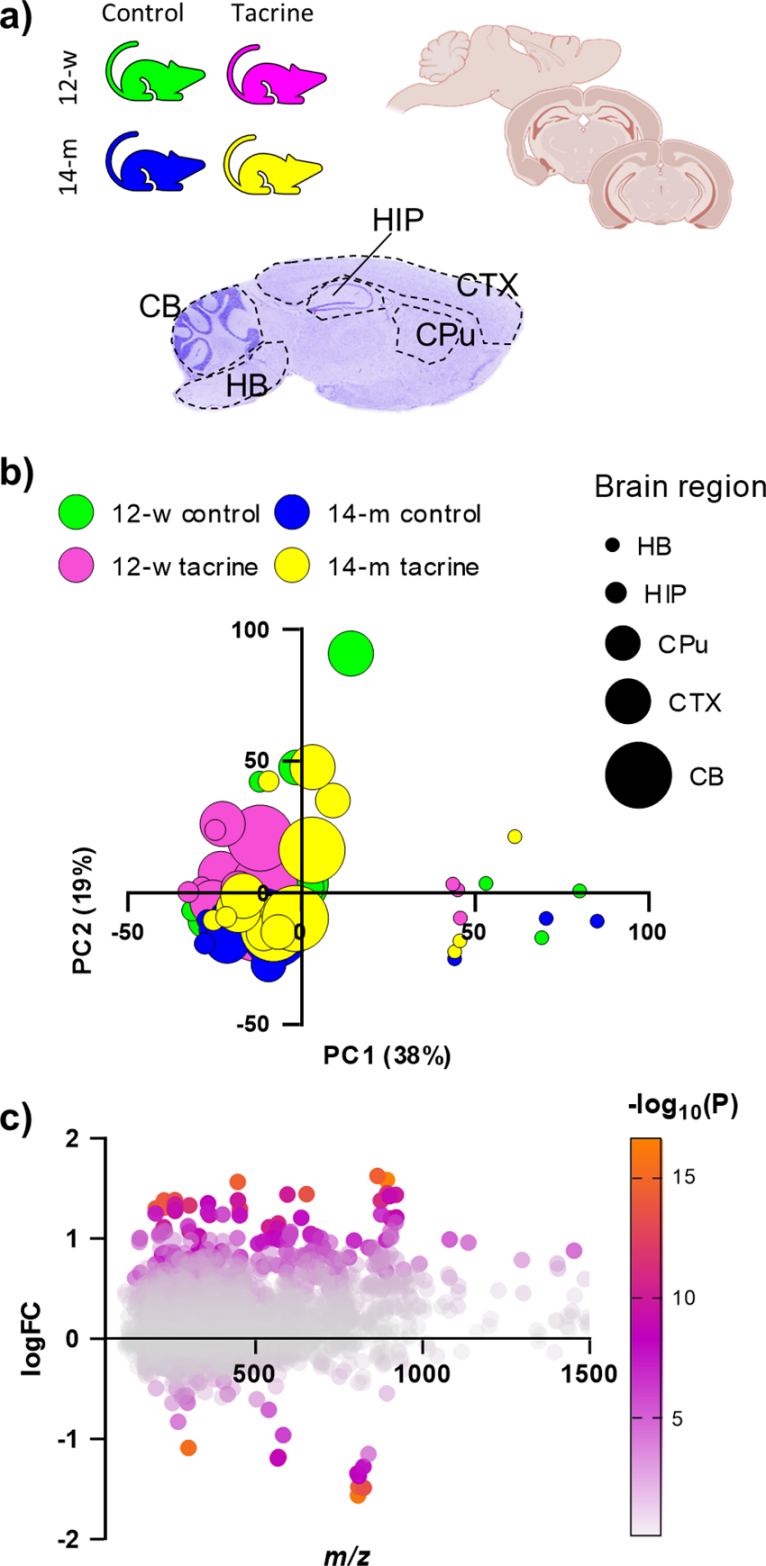
Overview of the spatial brain metabolomics analysis. (a) Graphical illustration of the investigated groups (*n* = 3) and the collected mouse brain tissue sections. The investigated brain regions are depicted on a Nissl-stained sagittal brain section. (b) Score values of the first (PC1) and second (PC2) principal components, as a result of principal component analysis. The parentheses following both components express the % of variance explained by each component. (c) Correlation plot for the log10 fold change (logFC) of detected features between the two age groups and the corresponding *m*/*z* value. The significance of the effect is illustrated using a color scale bar with the negative logarithmic *P* value. Abbreviations: CB, cerebellum; CPu, caudate putamen; CTX, cortex; HB, hindbrain; and HIP, hippocampus.

Age- and drug-induced neurochemical alterations in the mouse brain metabolome were investigated across four groups, i.e., 12-week-old naïve (12-w control) or tacrine-administered (12-w tacrine) mice, along with 14-month-old naïve (14-m control) or tacrine-administered (14-m tacrine) mice (Figure 1a). Mouse brain tissue sections were collected sagittally and coronally, the latter at different brain levels (Figure 1a). First, MALDI-MSI metabolomics analysis was performed on sagittal mouse brain tissue sections, with 9-amino acridine (9AA) serving as the MALDI matrix. Next, five different brain regions were selected for further investigation, i.e., the cortex (CTX), hippocampus (HIP), caudate-putamen (CPU), cerebellum (CB), and hindbrain (HB), including the medulla and pons (Figure 1a). The brain regions were selected based on our previous research on age-induced cholinergic and catecholaminergic changes, which highlighted the importance of the CTX, HIP, and CPU.^15,16^ In addition, we included CB and HB, which are rich in brain lipids, to explore potential new metabolic alterations. The regions were defined according to a mouse brain atlas.^21^ The whole brain (WB) was included as a reference.

Unsupervised principal component analysis (PCA) was performed to provide an overview of the data, providing information on the main sources of variance and identifying potential outliers. The PCA was based on extracted MS intensity values from five brain regions (CB, CPu, CTX, HB, HIP) of brain tissue sections from all four groups. The first principal component, PC1, revealed that the brain region exerts a large influence on the compounds detected (Figure 1b). This was further confirmed by a three-way ANOVA on the score values of PC1 (Figure S3, Tables S1, and S2). The unsupervised analysis revealed an age-treatment interaction effect in the CPu (Figure S3). Subsequently, the application of a linear model for age-induced alterations with covariate adjustments for treatment (tacrine) and brain regions indicated that approximately 17% of the total features, which covered a wide mass range, were significantly affected by age (Figure 1c). To highlight the regional effect of age and tacrine administration, a two-way ANOVA was applied to each region separately. This analysis revealed the highest metabolic impact of age on the CTX and HIP (Figure S4). This list of *m*/*z* values was further evaluated, with consideration given to brain distribution, peak shape, and intensity, to remove potential noise, isomers, and false positives; as a result, the final list represented 3% of the total features.

A complementary MSI analysis was performed in coronal mouse brain tissue sections. Whole brain average intensities were extracted to cross-validate the findings, which further limited the list of significant features to 2% of the original total features (Table S3). The levels of the coronal brain tissue sections were adjusted to sufficiently depict cortical and hippocampal regions, owing to the number of significant metabolic changes detected in these regions (Figure S4). In addition, higher lateral resolution MSI analyses, with *N*-naphthylethylenediamine dihydrochloride (NEDC) serving as the MALDI matrix, were performed to validate the results. The final results indicated that lipids, especially sulfatides, lysophosphatidic acids, and glutamine, are involved in age-associated mechanisms, including age-dependent tacrine effects (Table S3).

### Age-Induced Alterations of Brain Sulfatides

**Figure 2:**
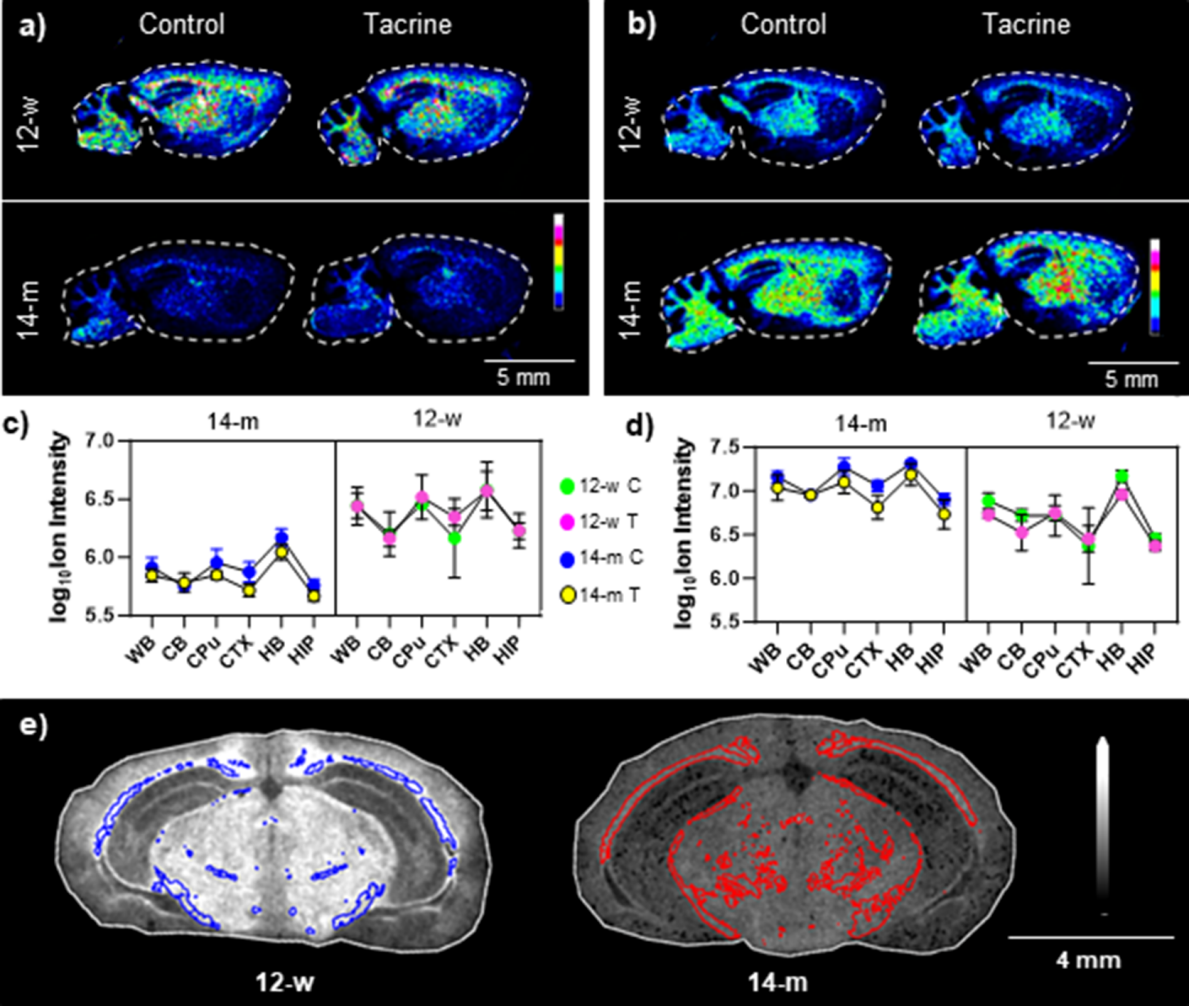
Age-induced alterations in sulfatides measured in the mouse brain. (a) Ion distribution image of the *m*/*z* 806.545 (C42, nonhydroxylated) in sagittal mouse brain tissue sections (lateral resolution: 100 μm, RMS normalization). (b) Ion distribution image of the *m*/*z* 904.618 (C48, hydroxylated) in sagittal mouse brain tissue sections (lateral resolution: 100 μm, RMS normalization); the ion intensities are scaled to 100% of total intensity. (c) Three-way ANOVA plot of the *m*/*z* 806.545 in the investigated brain regions of the two age (12-w and 14-m) and two treatment (control, C, and tacrine, T) groups. (d) Three-way ANOVA plot of the *m*/*z* 904.618 in the investigated brain regions of the two age (12-w and 14-m) and two treatment (control, C, and tacrine, T) groups, (*n* = 3). (e) Ion distribution image of the *m*/*z* 804.530 (C42, nonhydroxylated) in coronal mouse brain tissue sections (lateral resolution: 30 μm, TIC normalization, image scaled to 80% of total ion intensity). Segmentation-derived distinct classes are highlighted in blue (class 4) and red (class 5). Abbreviations: CB, cerebellum; CPu, caudate putamen; CTX, cortex; HB, hindbrain; HIP, hippocampus; and WB, whole brain.

A majority of the metabolites displaying significant age-induced alterations were identified as sulfatide species (SHexCer), including molecular ions as well as in-source induced product ions.^18,19^ Further structural validation was performed with DESI-MRM (Figure S5 and Table S3). Interestingly, age exerted differential effects on brain sulfatides depending on carbon chain length and hydroxylation status. In particular, hydroxylated species with sizable carbon chains, such as SHexCer(t42:2), were found to increase with age (Figure 2a–d and Table S3). In contrast, sulfatide species with shorter carbon chains and that are not hydroxylated, for instance, SHexCer(d36:1), were found to decrease with age (Figures 2a–d and S5). The results of segmentation analysis, an unsupervised spatial mapping method that creates pixel groups (classes) with similar spectra, also revealed that early aging significantly affects brain sulfatide levels. The segmentation analysis was performed on data collected at high lateral resolution (30 μm) using the timsTOF flex instrument. Although this analysis involved only one technical replicate per group, the high lateral resolution assisted the identification of age-specific classes in an unsupervised way. The analysis highlighted two significantly distinct classes, class 4 and class 5, which correspond to spectra from the 12-w and 14-m brain tissue sections, respectively, analyzed by MSI (class 4: 6322 spectra in 12-w control section, 4354 in 12-w tacrine region, 0 spectra in 14-m regions; class 5: 4320 spectra in 14-m control section, 4606 spectra in 14-m tacrine section, 0 spectra in 12-w sections). The derived classes 4 (blue) and 5 (red) demonstrated specific localization to white matter areas, i.e., corpus callosum and the thalamic and hypothalamic fibers, which provided detailed spatial information about the brain regions most affected by aging (Figure 2e). A receiver operating characteristic (ROC) analysis of classes 4 and 5, represented as two distinct regions, revealed a number of lipid species that contributed to the separation of the two classes (Figures 2e, S6, S7, and Table S4). The *m*/*z* 804.5299 ion species, which was found to decrease with age, was identified as SHexCer(d36:2) based on mass accuracy.

### Levels of Lysophosphatidic Acids Decrease with Age

**Figure 3:**
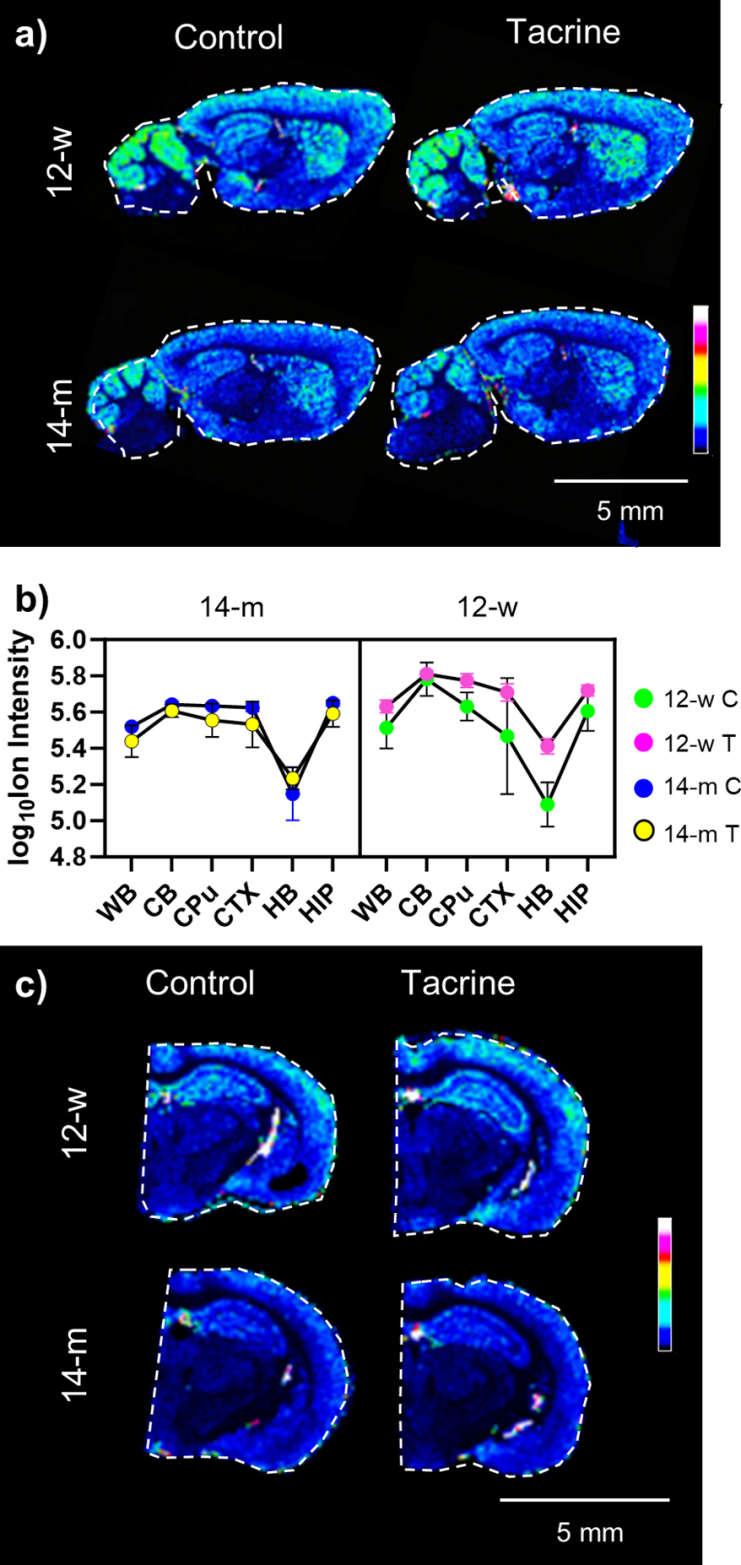
Age-induced alterations in lysophosphatidic acids measured in the mouse brain. (a) Ion distribution image of the *m*/*z* 415.226 in sagittal mouse brain tissue sections (lateral resolution: 100 μm); the ion intensities are scaled to 100% of total intensity. (b) Three-way ANOVA plot of the *m*/*z* 415.226 in the investigated brain regions of the two age (12-w and 14-m) and two treatment (control, C, and tacrine, T) groups (*n* = 3). (c) Ion distribution image of the *m*/*z* 415.226 in coronal half mouse brain tissue sections (lateral resolution: 100 μm); the ion intensities are scaled to 100% of total intensity. Abbreviations: CB, cerebellum; CPU, caudate putamen; CTX, cortex; HB, hindbrain; HIP, hippocampus; and WB, whole brain.

Our analyses revealed that two signals, i.e., *m*/*z* 415.226 and *m*/*z* 433.236, were detected at higher levels in 12-w mice—relative to other mice—and localized to gray matter regions, such as the cortex, striatum, and hippocampus (Figures 3 and S8). These signals correspond to two ion species of lysophosphatidic acid LPA(18:2), i.e., [M-H_2_O]^−^ and [M–H]^−^, as confirmed by the fatty acid fragment of FA(18:2) (*m*/*z* 279.232) in the MS/MS spectrum of *m*/*z* 415.226 (Figure S8). Also, it is notable that both species are commonly identified as fragments of larger lipids that contain FA(18:2).^22,21^ LPA(18:2) was found at significantly higher levels in the younger animals (12-week-old mice as compared to 14-month-old mice).

### Age- and Tacrine-Induced Alterations in the Glutamine/Glutamate Ratio (Gln/Glu)

**Figure 4:**
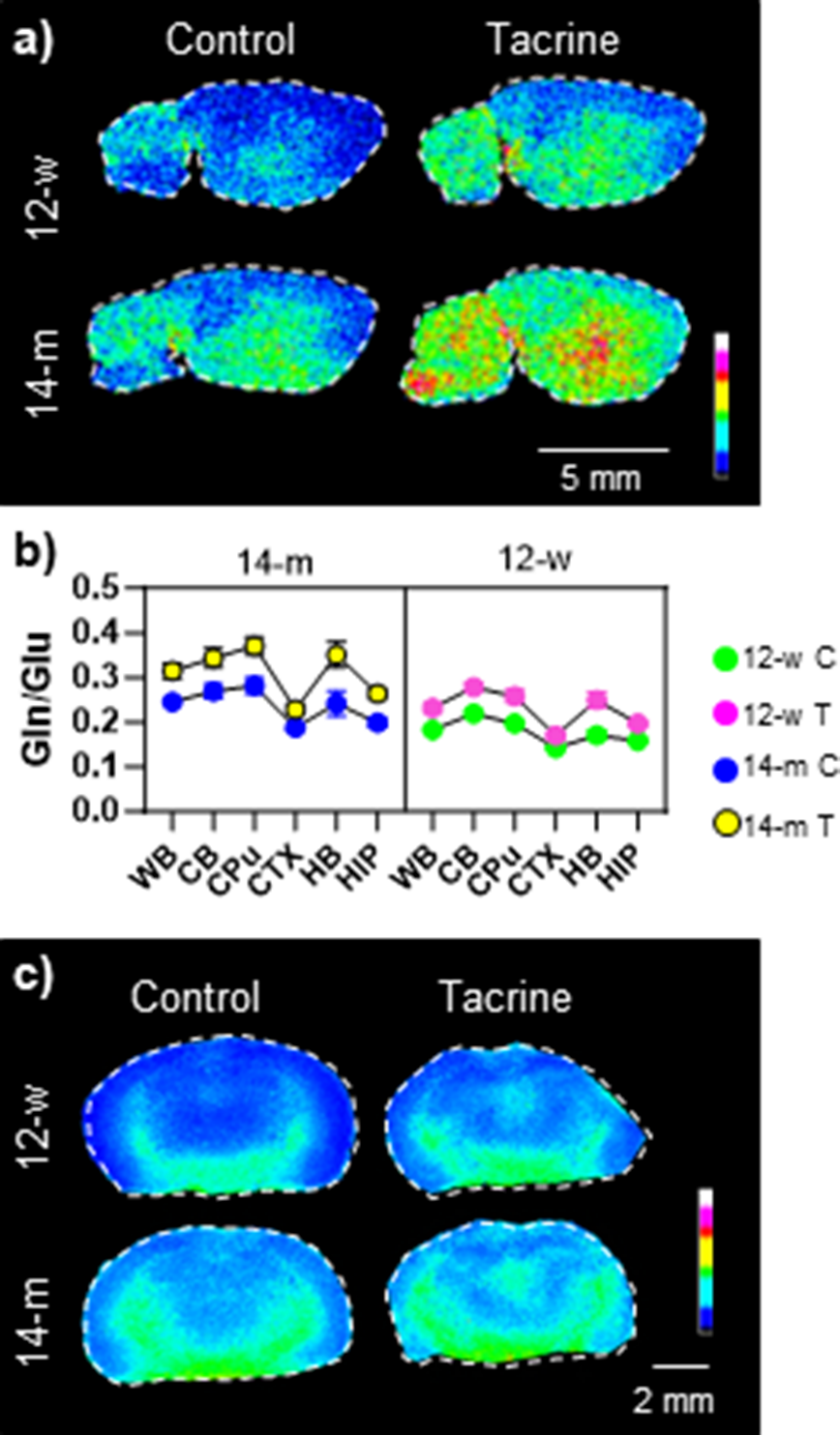
Age- and tacrine-induced alterations in the Gln/Glu ratio measured in the mouse brain. (a) Ion distribution image of the glutamine-to-glutamate ratio (Gln/Glu) in sagittal mouse brain tissue sections (9AA MALDI matrix; lateral resolution: 100 μm); the ion intensities are scaled to 100% of total intensity. (b) Three-way ANOVA plot of the Gln/Glu ratio in the investigated brain regions of the two age (12-w and 14-m) and two treatment (control, C, and tacrine, T) groups (*n* = 3). (c) MSI results regarding the glutamine-to-glutamate ratio (Gln/Glu) in coronal mouse brain tissue sections (NEDC MALDI matrix; lateral resolution: 70 μm); the ion intensities are scaled to 100% of total intensity. Abbreviations: CB, cerebellum; CPU, caudate putamen; CTX, cortex; HB, hindbrain; HIP, hippocampus; and WB, whole brain.

Tacrine administration resulted in an age-dependent increase in brain glutamine levels (Table S3). To further explore this dynamic, the glutamine/glutamate (Gln/Glu) ratio was used as an indicator of glia-neuronal function to limit the neurotoxic effects caused by excessive Glu levels.^23^ The older mice (14-m) showed significantly higher Gln/Glu ratios than the younger mice (12-y), with tacrine administration also increasing the Gln/Glu ratio (Figures 4 and S9). As the Gln/Glu ratio has been linked to glutamate neurotransmission, other Glu-derived metabolites that reportedly attenuate Glu neurotoxicity were examined. These included *N*-acetyl-aspartyl-glutamate (NAAG), which is the most abundant dipeptide in the brain that exhibits neurotransmitter functionality, and β-citryl-glutamate, an enigmatic pseudopeptide that was discovered in the newborn rat brain.^24−26^ Regarding NAAG, tacrine administration resulted in significant region-dependent effects, i.e., an interaction effect (Figures S10 and S11).

## Discussion

In the present study, spatial metabolomics was employed to highlight early aging-induced alterations in negatively charged small metabolites and lipids involved in metabolic pathways in the brain. Untargeted metabolic profiling, using high-mass resolution MALDI-FTICR imaging and high-lateral resolution MALDI-timsTOFflex imaging, underscored the significant impact of early aging on the levels of certain brain lipids, namely, sulfatides and LPAs. Additionally, metabolites located in cortical, hippocampal, and striatal regions were found to be significantly altered by age.

Sulfatides are glycosphingolipids that play a crucial role in maintaining the structural integrity of myelin.^27^ MSI studies have previously revealed regional changes in sulfatide levels in models of neurodegenerative diseases, i.e., AD and PD.^19,28^ It is also known that the composition of glycosphingolipids and glycerophospholipids within the brain changes during brain maturation/aging, with these alterations mainly affecting myelination.^27^ Here, we detected that the levels of certain sulfatide species fall with age, and this dynamic was particularly evident for species with low carbon chain lengths. In contrast, other sulfatide species, mainly hydroxylated analogues with longer carbon chains, demonstrated elevated levels during aging. Altered sulfatide metabolism has previously been linked to oxidative stress and demyelination,^19^ both of which are pathophysiological mechanisms involved in aging. The differential effects of aging observed in the present study, i.e., the impact of carbon chain length and hydroxylation status, reflect the distinct functionalities and cellular distributions of different sulfatide species.^19^

Another lipid class, LPAs, demonstrated age-induced changes in the analyzed mouse brains. LPA(18:2) was found at significantly higher levels in younger animals (i.e., 12-w vs 14-m), especially in the striatal area and the hippocampus. This may indicate that LPAs are involved in neuroplasticity and growth; notably, a clear correlation between LPA plasma levels and mild cognitive impairment has been reported in humans.^29^

The glutamate–glutamine cycle is crucial for regulating the levels of glutamate in the brain, as this neurotransmitter can have excitotoxic effects when present at excessive levels. During glutamate metabolism, which is coordinated by neuron-astrocyte cooperation,^23,29−31^ glutamate taken up from the extra-synaptic space is converted into glutamine, which can be reabsorbed by the presynaptic neuron. Glutamine, once it is transported inside the neuron, will be converted into glutamate, which completes the glutamate turnover cycle.^23,32^ In the present study, both aging and tacrine administration were found to elevate the Gln/Glu ratio, which is indicative of an imbalance in the neural-astroglial regulatory mechanism.^33^ In addition, it is important to note that tacrine has demonstrated neuroprotective effects against glutamate neurotoxicity in cerebral cortex cell cultures.^34^ Since the conversion of glutamate to derivatives such as NAAG and β-citryl glutamate can also exert neuroprotective effects, we investigated the brain distribution of these modified peptides and found specific localization to midbrain regions.

The presented results complement the aforementioned findings of how aging induces region-specific changes in the brain metabolome by providing structural validation and brain mapping of multiple relevant metabolites. Therefore, our approach can assist and guide further spatial metabolomics studies on brain aging and other neuropathological conditions.

## Methods

### Chemicals

The solvents, water, methanol, and acetonitrile used in the experiments were of HPLC grade (VWR, Radnor, PA, USA). 9-Aminoacridine (9AA), *N*-naphthylethylenediamine dihydrochloride (NEDC), *N*-acetyl-aspartyl-glutamate (NAAG), l-glutamate, l-glutamine, L-aspartate, taurine, and glutathione were purchased from Sigma-Aldrich (St. Louis, MO, USA).

### Animal Experiments

Male mice (C57BL/6J) 12 weeks (12-w, *n* = 8) or 14 months (14-m, *n* = 8) of age were obtained from Janvier laboratories (Scand-LAS, Turku, Finland). The animals were housed under controlled temperature and humidity (20 °C, 53% humidity) under a 12 h light/dark cycle and fed ad libitum. All of the experiments were carried out in accordance with European Council Directive 86/609/EEC and approved by the local Animal Ethical Committee (approval no. N40/13 and N275-15). Tacrine was dissolved in saline and administered intraperitoneally (i.p.) at a dose of 10 mg/kg to both the 12-w and 14-m mice. Control animals were injected with an equivalent amount of saline solution. Animals were euthanized 30 min after injection by decapitation, after which the brains were rapidly dissected out, snap-frozen in cold isopentane, and stored at −80 °C.

### Tissue Processing and Sample Preparation

Tissue sectioning was performed at −20 °C using a CM1900 UV cryostat-microtome (Leica Microsystems, Wetzlar, Germany). Coronal and sagittal brain tissue sections were cut at a thickness of 12 μm and subsequently thaw-mounted on conductive indium tin oxide-coated glass slides (Bruker Daltonics, Bremen, Germany), or on regular slides for DESI-MRM. Three brain tissues from each examined group were first sectioned sagittally, approximately until the midline, and then sectioned coronally. This approach allowed better visualization of multiple brain regions from the same animal. Additionally, one brain tissue from each group was only sectioned coronally for complete visualization of the metabolic changes. Three brain tissue sections (biological replicates) per group were analyzed both sagittally and coronally. The prepared slides were stored at −80 °C. Prior to imaging, the sections were desiccated at room temperature for 15 min, after which optical images were captured using a photo scanner (Epson Perfection V500, Nagano, Japan).

The MALDI-MSI matrices (9AA and NEDC) were applied with an automatic TM sprayer (HTX-Technologies LLC, Chapel Hill, NC, USA). The following parameters were used for the 9AA (5 mg/mL dissolved in 80% methanol) application: 75 °C, six passes, the solvent flow rate of 70 μL/min, spray head velocity of 1100 mm/min, and track spacing of 2.0 mm. The following parameters were used for NEDC (7 mg/mL dissolved in 70% methanol) application: 50 °C, 16 passes, solvent flow rate of 70 μL/min, spray head velocity of 1100 mm/min, and track spacing of 2.0 mm. N_2_ gas pressure was always set at 6 psi.

### MALDI-MSI Analysis

For the spatial metabolomics analysis, the MALDI-MSI experiments were performed in negative ionization mode using a MALDI-FTICR (Solarix XR 7T-2Ω, Bruker Daltonics) mass spectrometer, which was chosen due to the ultrahigh mass resolution and mass accuracy. The FTICR instrument was equipped with a Smartbeam II 2 kHz laser. The instrumental setup was optimized for the detection of small molecules (approximately m/z 80−1000, depending on the applied MALDI matrix) when using the quadrature phase detection (QPD) (2ω) mode. For the 9AA-coated mouse brain tissue sections, the medium laser focus setting was used at a frequency of 1 kHz, and laser power was optimized prior to acquisition. Spectra were collected by summing signals from 100 laser shots per pixel. The quadrupole isolation *m*/*z* (Q1) was set at *m*/*z* 120. The TOF and transfer optics frequency values were adjusted to 0.650 ms and 4 MHz, respectively. A matrix-derived peak at *m*/*z* 193.077122 was used as a lock mass for internal *m*/*z* calibration. The same laser settings were applied for the NEDC-coated mouse brain tissue sections, while Q1 was set at *m*/*z* 80 and the TOF and transfer optics frequency values were adjusted to 0.550 ms and 6 MHz, respectively. Red phosphorus was used for the external calibration of the method across all experiments. Samples were analyzed in a random order to prevent bias arising from matrix vacuum instability or changes in mass spectrometer sensitivity.

Higher lateral resolution experiments (30 μm) were performed using a timsTOF flex instrument (Bruker Daltonics) which integrates high-speed and high-lateral resolution MALDI imaging with ion mobility mass spectrometry. The NEDC MALDI matrix was applied to coronal mouse brain tissue sections collected from all of the investigated groups (one mouse brain tissue per group). MSI data were acquired in negative ionization mode at *m*/*z* range of 80–1600. Prior to the MSI experiment, the slides underwent height correction and focus adjustment. Following method evaluation, no post-ionization or trapped ion mobility spectrometry (TIMS) was selected. Spectra were collected by summing signals from 100 laser shots per pixel with a laser power of 58% and a frequency of 10 kHz. Sweeping mode was implemented to increase the sensitivity of the analysis toward multiple mass ranges, e.g., switching twice between collision RF 700 Vpp/transfer time 50 μs and collision RF 2500 Vpp/transfer time 100 μs for every pixel.

For tissue samples and standards, when available, MALDI-MS/MS experiments were performed by isolating the precursor ion in a mass window of 1 or 2 Da and allowing the target ions to be selected in the quadrupole and fragmented in the collision cell. The collision energy, which varied between 5.0 and 35.0 V, was optimized for every analyte. Following MALDI-MSI analysis, the sections were histologically analyzed using Nissl staining.

### Imaging Analysis

The MSI data were visualized in FlexImaging (v. 5.0, Bruker Daltonics). When further analysis was needed, the data were imported into SCiLS Lab (v. 2023b Pro, Bruker Daltonics), and brain regions were annotated according to a stereotaxic atlas.^34^ The analyses included all four experimental groups: 12-w control; 14-m control; 12-w tacrine-treated; and 14-m tacrine-treated. The initial evaluation of sagittal sections of brain tissue identified five anatomically distinct brain regions for further investigation: the cortex (CTX); hippocampus (HIP); caudate-putamen (CPU); cerebellum (CB); and hindbrain (HB). The coronal sections obtained from the extracted whole brains were also analyzed. All individual spectra were normalized to the root-mean-square (RMS) value calculated from all of the data points. The maximum ion intensities for the 2500 most intense peaks within the average spectra for each brain region in the mass range *m*/*z* 107–1000 (9AA experiments) or *m*/*z* 86–1000 (NEDC experiments) were exported from SCiLS for statistical analysis. The average intensity values per brain area were log10 transformed.

### Data Analysis

The regional data from the sagittal sections were analyzed first. Multivariate analyses were performed in SIMCA v.17.0 (Sartorius Stedim Biotech, Umeå, Sweden). Because all of the included variables were measured in the same unit, i.e., log10-transformed ion intensities, centering and autoscaling to unit variance (the SIMCA default scaling option) were considered adequate. PCA was initially applied to obtain an overview of the data and identify possible outliers. The Hotelling T2 ellipse (T2Crit) and distance to model (DModX) at the 95% confidence interval were used as criteria for outlier detection.

A multivariable two-way ANOVA (two-level factors: age, treatment) with false discovery rate (FDR) correction for multiple tests was performed separately for each brain region using the open-source statistical tool metaboanalyst^35^ (www.metaboanalyst.ca). Linear models with covariate adjustments were applied to cross-validate the results. The underlying method is based on limma due to high-performance implementation.^36^ A three-way ANOVA (factors: age, treatment, brain regions) was performed for selected and identified metabolites using GraphPad Prism 9.

### Segmentation Analysis

Unsupervised spatial segmentation analysis was performed in SCiLS Lab software using the data acquired at 30 μm via the timsTOF flex instrument. Prior to analysis, the data were normalized to total ion count. The input feature list included all of the most abundant acquired m/z values (1468) and segmentation was performed on all of the individual spectra collected from the analyzed brain tissue sections. In this analysis, bisecting *k*-means with correlation distance as a metric was applied. This analysis provides a map in which every spectrum is assigned a label; this creates groups that share similar spectra, with the results reported as label objects. To identify the features leading to the clustering, ROC analysis was consequently performed with an area under the ROC curve (AUC) threshold of AUC > 0.75 or AUC < 0.25.

### Identification of Metabolites

The ions that displayed significant age- or tacrine-related alterations were primarily identified by database searches (www.hmdb.ca,^17^www.lipidmaps.org,^20^ and metaspace2020.eu^37^) based on the high mass accuracy provided by the FTICR MS analysis. Standards were also used to confirm the identities of detected ions. MALDI-MS/MS was performed on tissue sections and the product ions were compared to the product ion spectra of standards or previously published data. In the case of MS/MS imaging, the brain tissue distributions of product ions were compared to the distribution of the precursor ion. For small *m*/*z* species that were colocalized with sulfatide species, yet had insufficient tissue abundance to perform MS/MS, molecular formulas were assigned with the assumption that the species contains an S atom (Smart Formula, Bruker Daltonics).

DESI experiments were performed using a XevoTM TQ-XS triple quadrupole mass spectrometer (Waters Corporation, Manchester, UK) equipped with a two-dimensional DESI XS source containing a high-performance DESI sprayer and heated transfer line (Waters Corporation, Manchester, UK); the experiments were performed in tandem-MS and MRM mode. The DESI solvent, composed of methanol/water (MeOH/H2O) 95:5 (v/v), was delivered using a single syringe infusion pump (KD Scientific, Holliston, MA, USA) at a flow rate of 2 μL/min and nitrogen gas at an optimized nebulizing gas pressure of 10 psi. The spray was delivered at a voltage of 0.6 kV and a cone voltage of 20. The heated transfer line was set to a temperature of 450 °C to increase the lipid signal. DESI-tandem-MS, in negative ionization mode, was performed on tissue sections at CID 20–55% for sulfatide species ST C24:1 (*m*/*z* 888.6, C48H90NO11S). DESI-MRM was performed on tissue sections for *m*/*z* values of 804.5, 806.5, 822.5, 837.5, 863.6, 874.6, 888.6, 891.6, 892.6, and 904.6 at CID 55%, with the fragment ion at *m*/*z* 97 for all transitions corresponding to the characteristic sulfatide fragment HO4S-. DESI images were acquired at 100 × 100 μm/pixel and 5 scans/s, resulting in a constant speed of 500 μm/s in the *X* direction. The imaging data were visualized using High-Definition Imaging (HDI) 1.6 (Waters Corporation).

## Supporting Information Available

Detection and identification of brain metabolites with MALDI-MSI, identification of brain metabolites by tandem MS in negative ionization mode, evaluation of the impact of brain region, age, and tacrine administration on the first principal component using three-way ANOVA, Venn diagram of the significant features per brain region using two-way ANOVA, mass spectrometry imaging and identification of brain sulfatides, segmentation and ROC analysis revealed age-associated lipids at high lateral resolution, ROC curve visualizing the discrimination capability of two features highlighted by the segmentation analysis, age-induced alterations of lysophosphatidic acids in the mouse brain, Box plot of the log_10_ Gln/Glu ratio in coronal brain tissue sections, MSI of glutamate derivatives on brain tissue sections, structural validation of NAAG with MS/MS imaging using a standard and a brain tissue section, three-way ANOVA on the impact of brain region, age and tacrine administration on the first principal component, multiple comparisons of the PC1 score values among different groups and brain regions, with Tukey’s correction, and list of significantly altered features based on limma analysis (PDF)
