# Supplementary material for: Applying Spatial Metabolomics To Investigate Age- and Drug-Induced Neurochemical Changes
Source: ACS Chem Neurosci. 2024 Jul 29;15(15):2822–9. doi: 10.1021/acschemneuro.4c00199 (PMC11311129; doi:10.1021/acschemneuro.4c00199)
Supplement: Supplementary file 1 — cn4c00199_si_001.pdf [file cn4c00199_si_001.pdf]

Supporting Information

Applying spatial metabolomics to investigate age- and drug-induced neurochemical changes

Theodosia Vallianatou[1*], Tina B. Angerer[1], Ibrahim Kaya[1], Anna Nilsson[1], Reza Shariatgorji[1], Per Svenningsson[2] and Per E. Andrén[1*]

1 . Dept. of Pharmaceutical Biosciences, Spatial Mass Spectrometry, Science for Life Laboratory, Uppsala University, SE-75124 Uppsala, Sweden.

2 . Dept. of Clinical Neuroscience, Karolinska Institute, SE-17177 Stockholm, Sweden.

*Corresponding authors. Emails: theodosia.vallianatou@uu.se, per.andren@uu.se

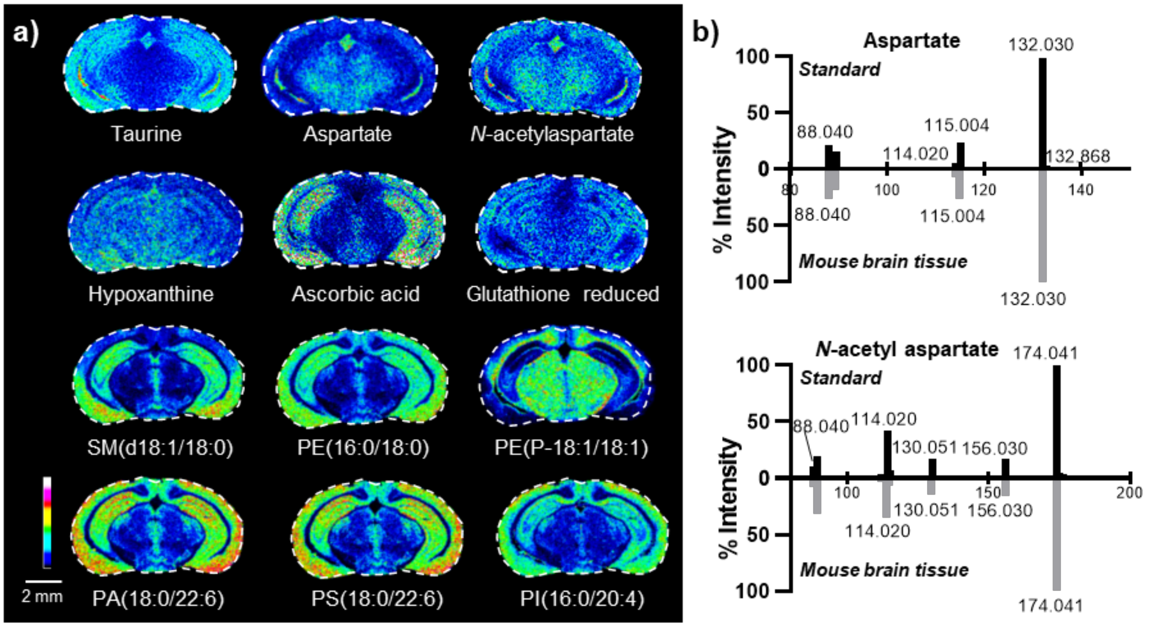

Figure S1. Detection and identification of brain metabolites with MALDI-MSI. (a) Several brain metabolites detected using the MSI method with the 9AA MALDI matrix. The distribution of these metabolites is illustrated across a coronal mouse brain tissue section at 60 µm lateral resolution. All images are normalized to RMS and scaled to 50% of maximum intensity. (b) Representative tandem MS spectra (*m/z* values extracted at 1.5 signal-to-noise ratio) collected from reference standards and mouse brain tissue sections (5 V) for metabolite identification at the maximum confidence level.

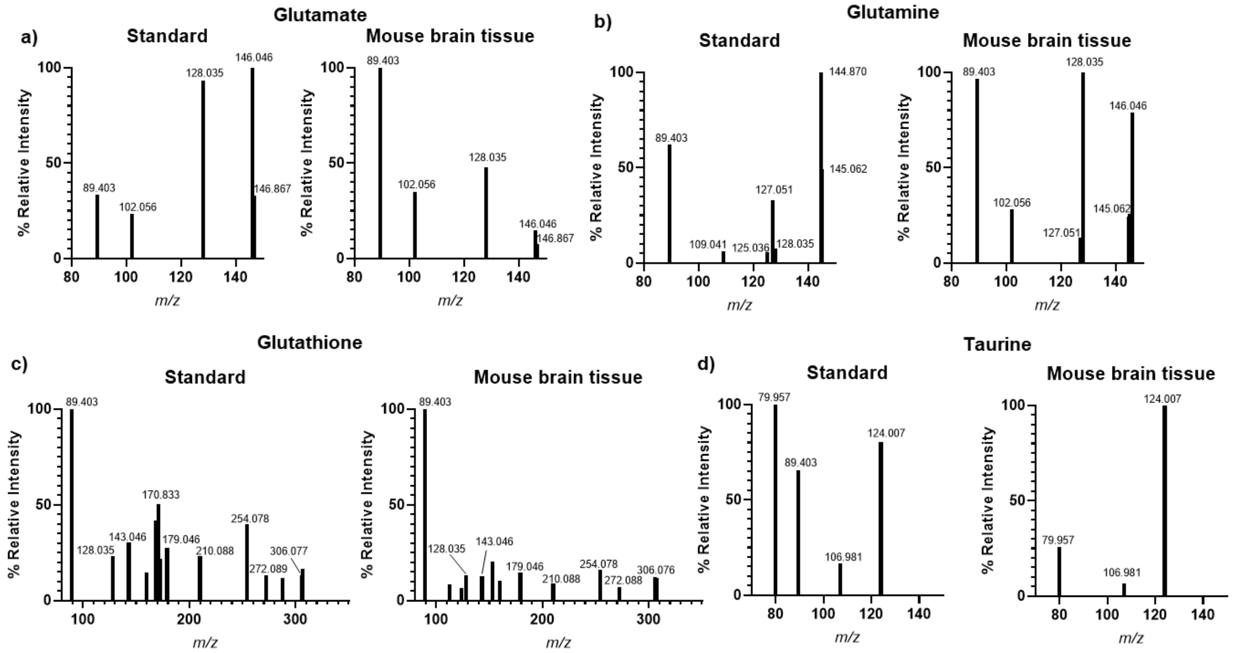

Figure S2. Identification of brain metabolites by tandem MS in negative ionization mode. (a) MS/MS spectra of glutamate acquired for the reference standard (left) and brain tissue sections (right) at 5 V. (b) MS/MS spectra of glutamine acquired for the reference standard (left) and brain tissue sections (right) at 5 V. (c) MS/MS spectra of glutathione acquired for the reference standard (left) and brain tissue sections (right) at 10 V. (d) MS/MS spectra of taurine acquired for the reference standard (left) and brain tissue sections (right) at 15 V. The *m/z* values were extracted at a 1.5 signal-to-noise ratio.

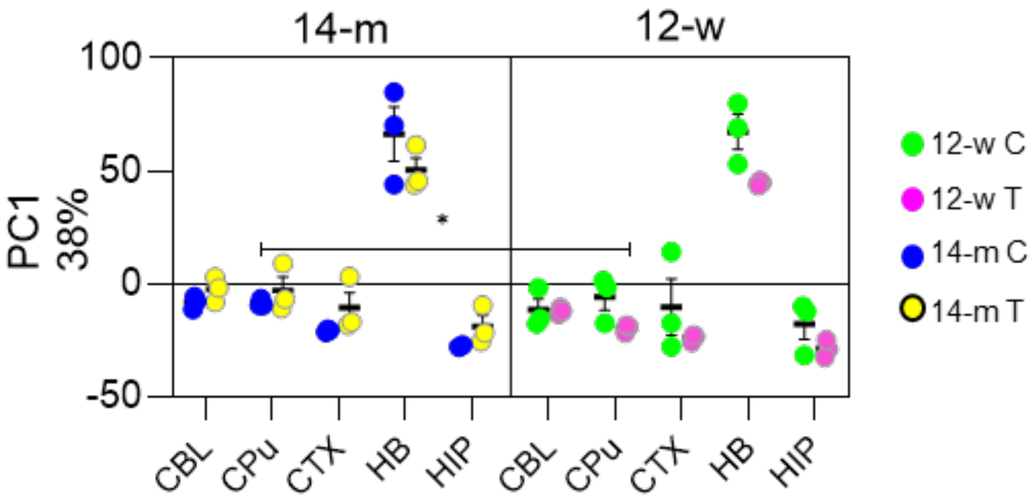

Figure S3. Evaluation of the impact of brain region, age and tacrine administration on the first principal component using three-way ANOVA. Score values of the first principal component (PC1), explaining 38% of the total variance, are plotted according to brain region, age and treatment. A detailed report of the analysis is provided in Tables S1 and S2. The region demonstrating significant differences in score values between the different examined groups is the caudate-putamen (CPu). *P < 0.05. Abbreviations: CB, cerebellum; CPu, caudate putamen; CTX, cortex; HB, hindbrain; HIP, hippocampus.

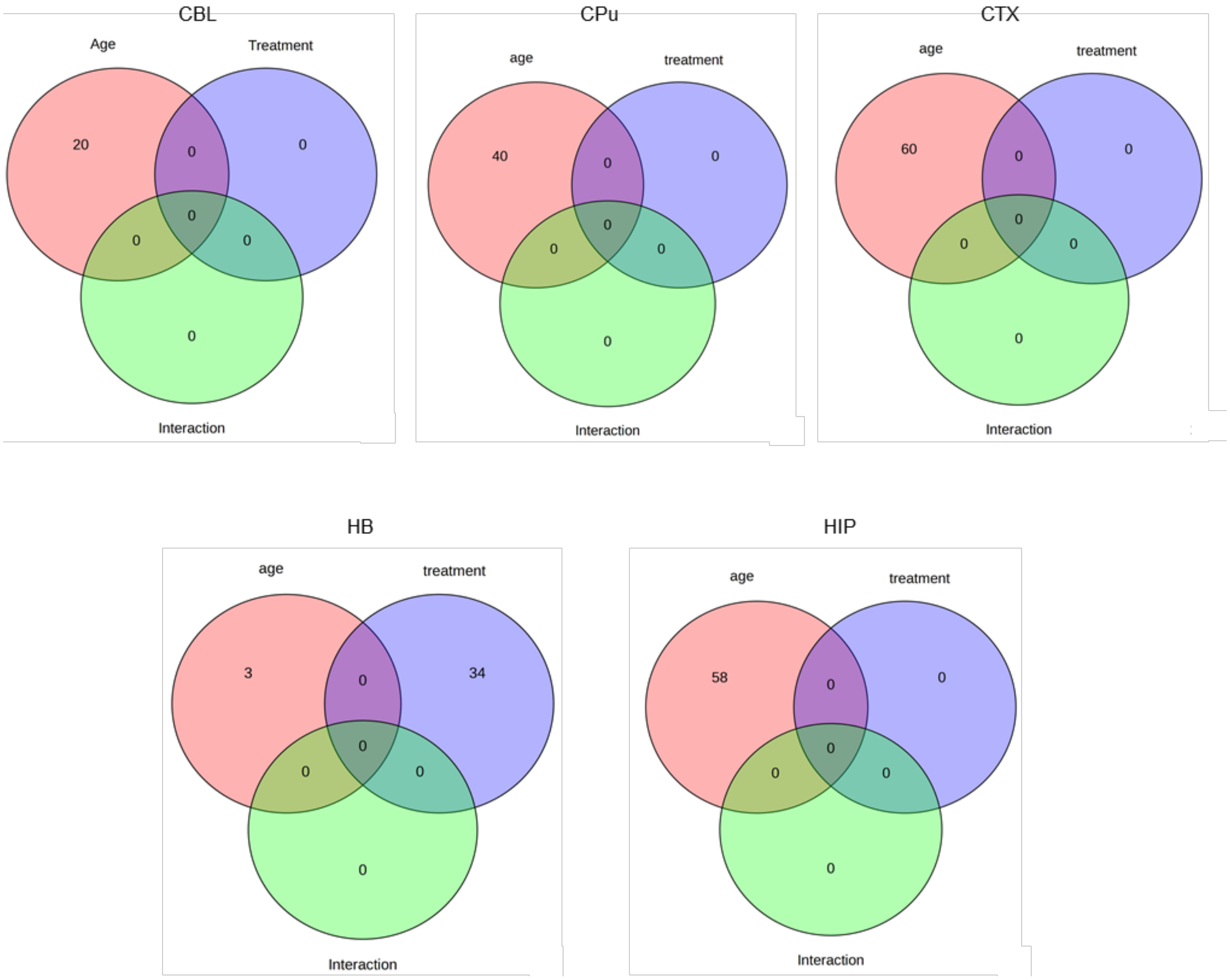

Figure S4. Venn diagram of the significant features per brain region using two-way ANOVA (based on the top 200 features). Abbreviations: CB, cerebellum; CPu, caudate putamen; CTX, cortex; HB, hind-brain; HIP, hippocampus

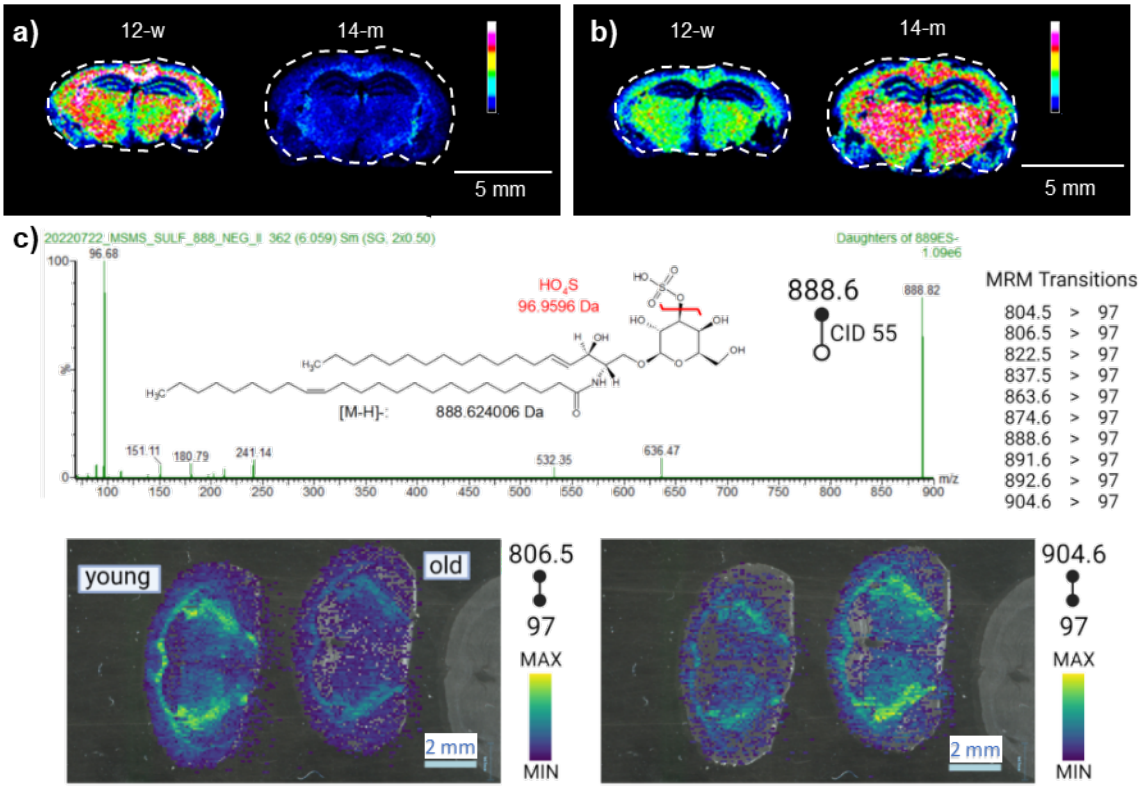

Figure S5. Mass spectrometry imaging and identification of brain sulfatides. (a) Ion distribution image of *m/z* 806.545 in coronal mouse brain tissue sections (lateral resolution: 100 µm). **(**b) Ion distribution image of *m/z* 904.618 in coronal mouse brain tissue sections (lateral resolution: 100 µm). The ion intensities are scaled to 50% of total intensity. (c) Structural validation of sulfatides by DESI MRM and MSI of the characteristic product ion with *m/z* 97.

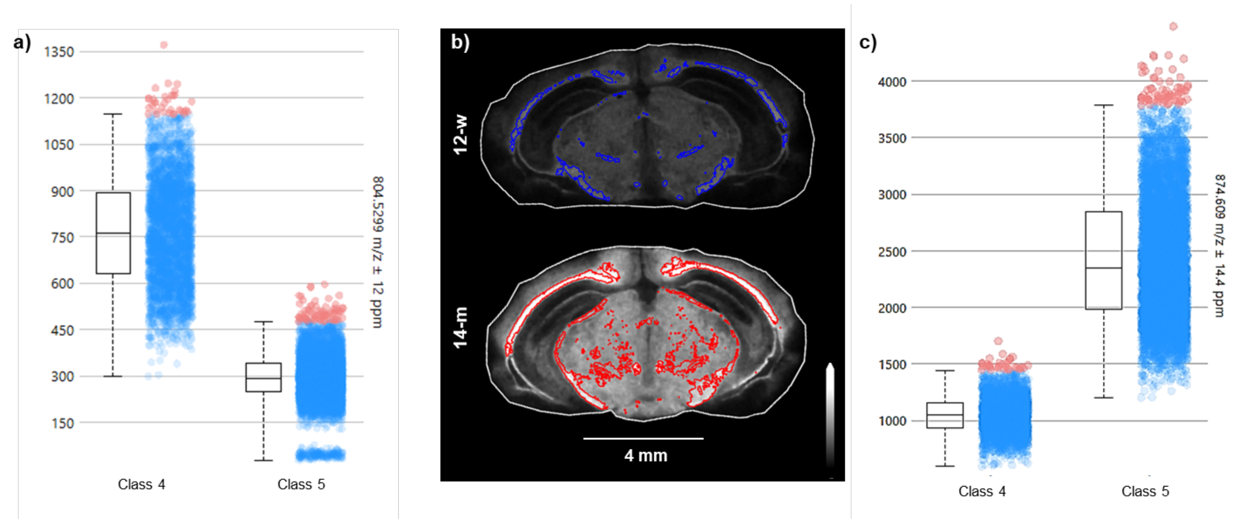

Figure S6. Segmentation and ROC analysis revealed age-associated lipids at higher lateral resolution. (a) Intensity box plot of *m/z* 804.530 for the two segmentation classes. (b) Ion distribution image of *m/z* 874.609 in coronal mouse brain tissue sections from 12-week-old and 14-month-old animals, respectively (lateral resolution: 30 µm, TIC normalization). Segmentation-derived distinct classes are highlighted in blue (class 4) and red (class 5). (c) Intensity box plot of *m/z* 874.609 for the two segmentation classes (lateral resolution: 30 µm).

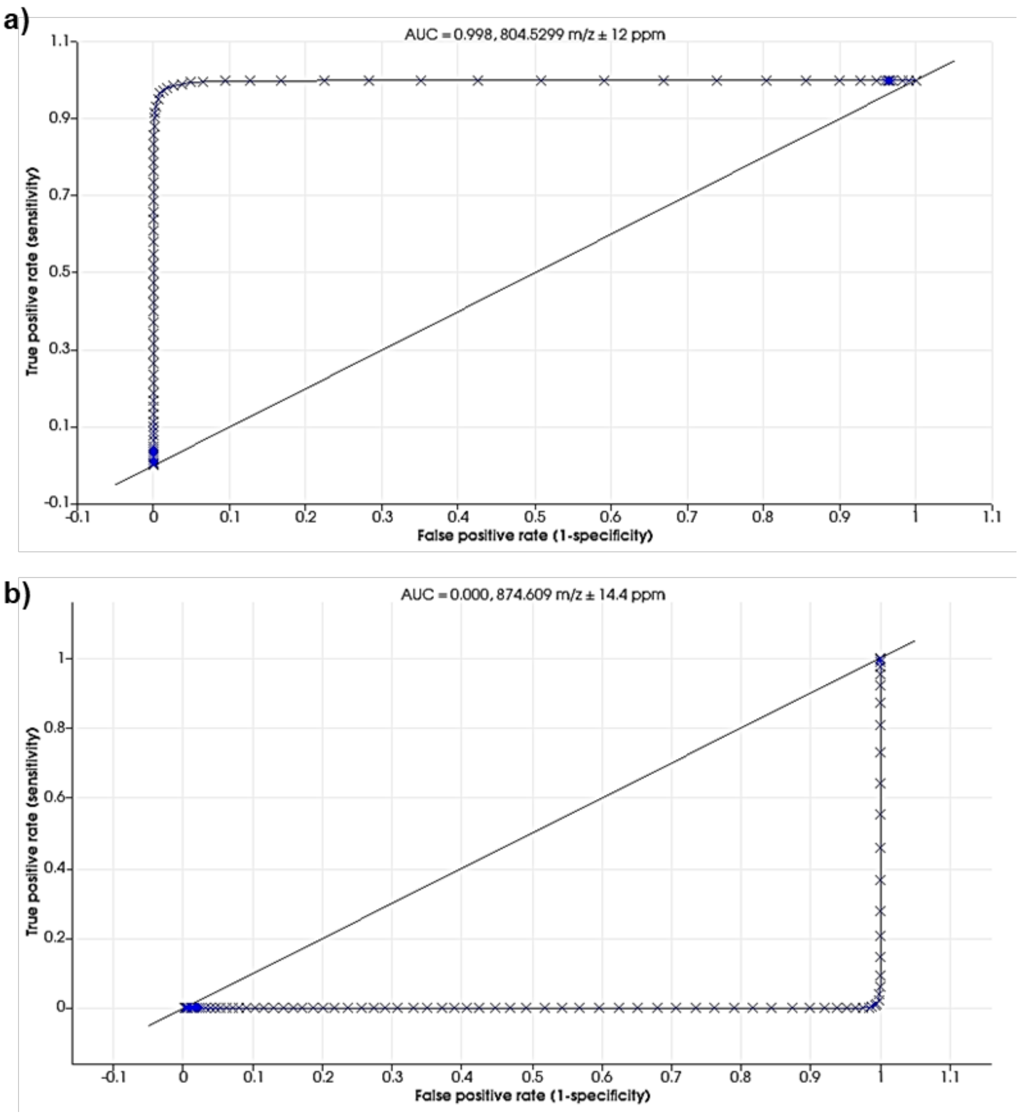

Figure S7. ROC curve visualizing the discrimination capability of two features highlighted by the segmentation analysis. (a) ROC curve for *m/z* 804.5299, which is elevated in segmentation class 4 (12-w). (b) ROC curve for *m/z* 874.609, which is elevated in segmentation class 5 (14-m). The area under the ROC curve (AUC) measures the discrimination quality across the interval 0.0 to 1.0, with perfect discrimination giving an AUC value of exactly 1 or 0; the AUC values are specified in graphs (a) and (b).

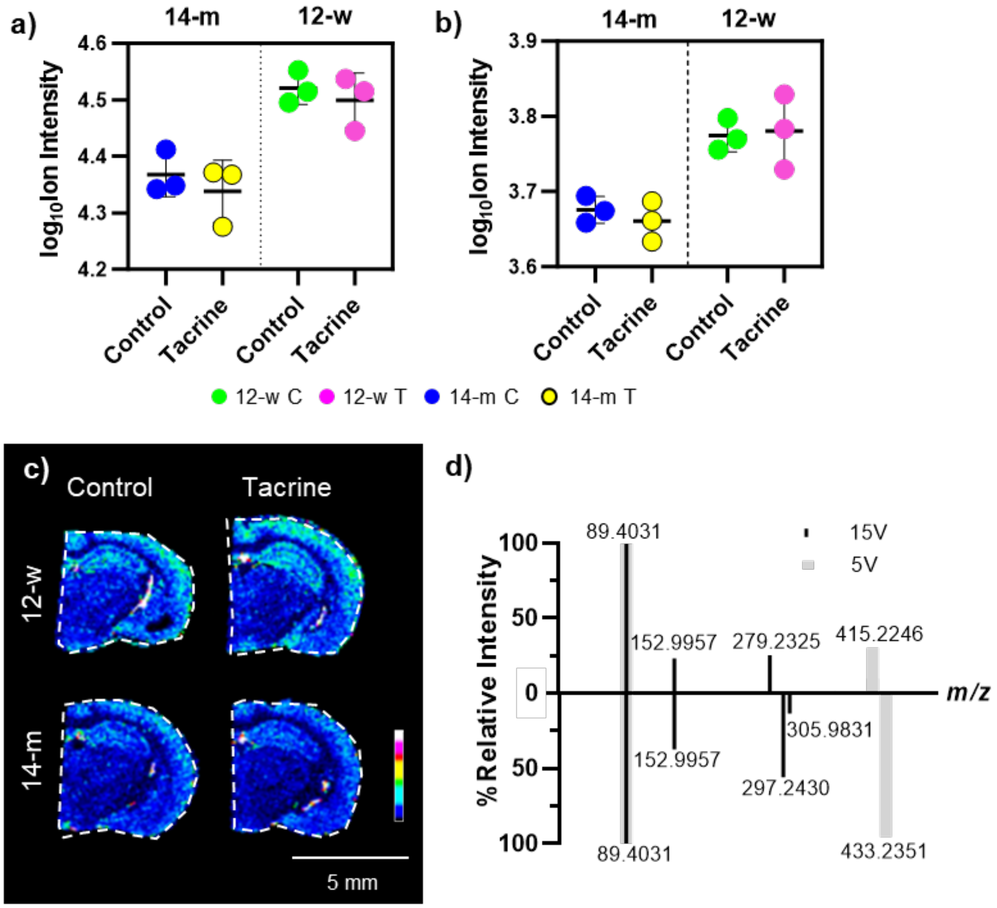

Figure S8. Age-induced alterations of lysophosphatidic acids in the mouse brain. (a) Dot plots of the log-transformed ion intensities of *m/z* 415.226 in coronal brain tissue sections. (b) Dot plots of the log-transformed ion intensities of *m/z* 433.236 in coronal brain tissue sections. (c) Ion distribution image of *m/z* 433.236 in coronal half mouse brain tissue sections (lateral resolution: 100 µm). The ion intensities are scaled to 100% of total intensity. (d) MS/MS spectra of the precursor ions *m/z* 415.226 (upper spectrum) and *m/z* 433.236 (lower spectrum) in coronal mouse brain tissue sections using two different collision energies, 5V and 15V. Peaks were extracted with a 1.5 S/N ratio.

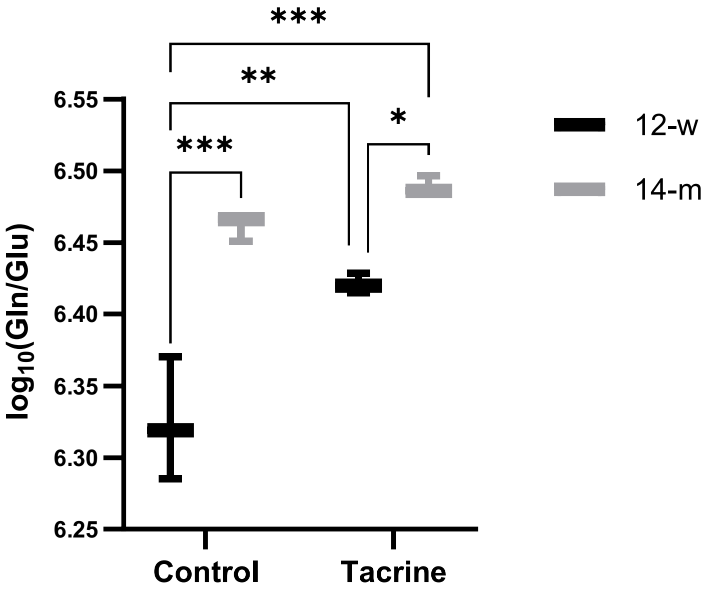

Figure S9. Box plot of the $\log_{10}$ Gln/Glu ratio in coronal brain tissue sections. Two-way ANOVA plot of the $\log_{10}$ Gln/Glu ratio in whole brain tissue sections of the two age (12-w and 14-m) and two treatment (control and tacrine) groups (n=3). The Gln/Glu ratio increased with age and tacrine administration. *P<0.05, **P<0.01, ***P<0.001

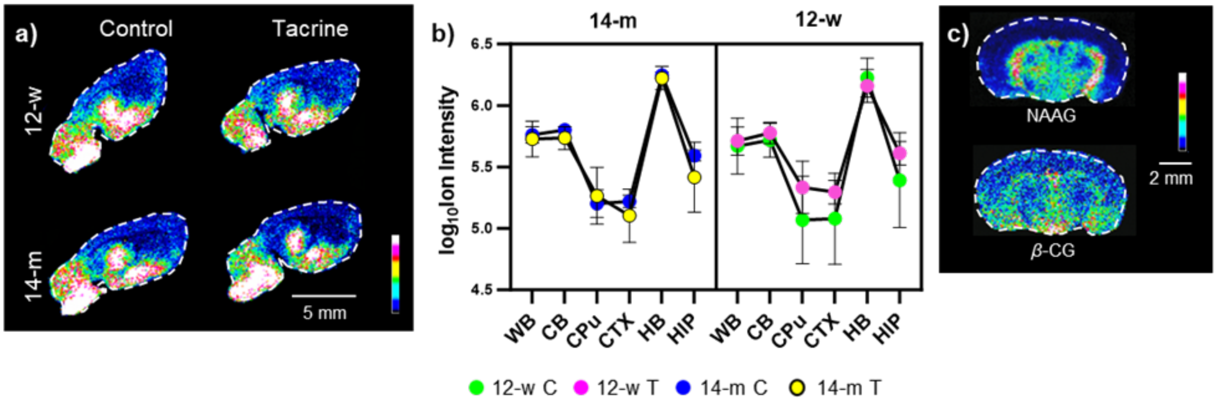

Figure S10. MSI of glutamate derivatives on brain tissue sections. (a) Ion distribution image of *N*-acetyl-aspartyl-glutamate (NAAG) in sagittal mouse brain tissue sections when 9AA MALDI served as the matrix (lateral resolution: 100 µm). (b) Three-way ANOVA plot of NAAG in the investigated brain regions of the two age (12-w and 14-m) and two treatment (control and tacrine) groups. (c) Ion distribution images of NAAG (upper section) and *β*-citryl-glutamate (lower section) in a coronal mouse brain tissue section when 9AA MALDI served as the matrix (lateral resolution: 60 µm). Significance of the three investigated factors and their interactions: brain region, ****P < 0.0001; age, ns; treatment, ns; region x age, ns; region x treatment, *P=0.045; age x treatment, ns; region x age x treatment, *P=0.015. The ion intensities are scaled to 50% of total intensity. Abbreviations: CB, cerebellum; CPu, caudate putamen; CTX, cortex; HB, hindbrain; HIP, hippocampus, WB, whole brain.

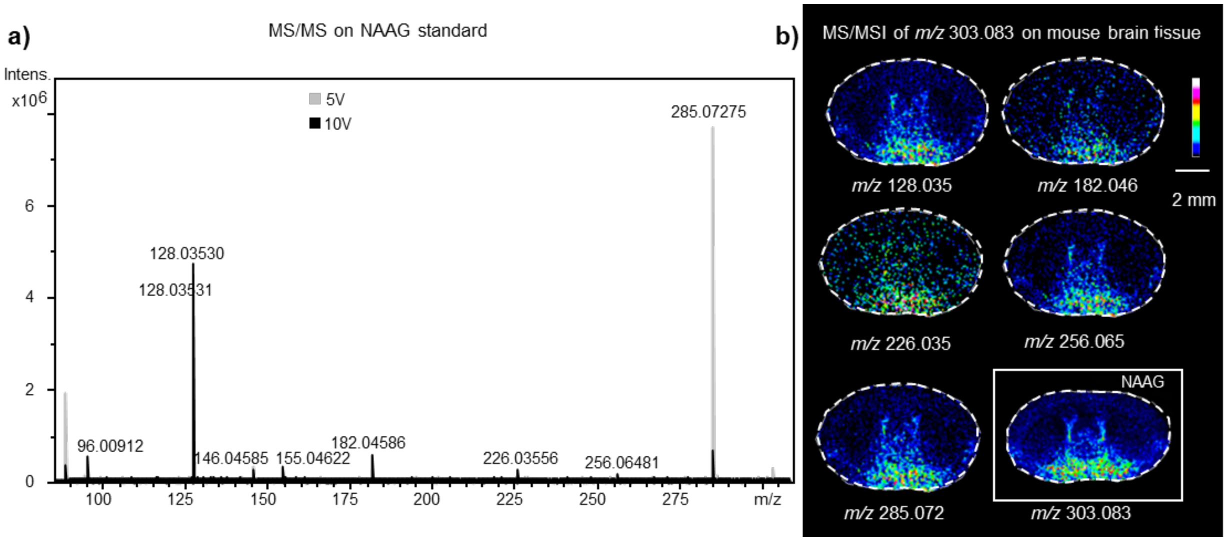

Figure S11. Structural validation of NAAG with MS/MS imaging using a standard and a brain tissue section. (a) Overlaid MS/MS of the precursor ion *m/z* 303.083 acquired with 5V (grey) and 10 V (black) from a reference standard of *N*-acetyl-aspartyl-glutamate (NAAG). (b) MS/MS imaging of the precursor ion *m/z* 303.083 on coronal mouse brain tissue sections. The highlighted product ions were detected in the reference standard of NAAG. Peaks were extracted with a 1.5 S/N ratio.

Table S1. Three-way ANOVA on the impact of brain region, age and tacrine administration on the first principal component.

Source of Variation	% of total variation	P value	P value summary	Significant
Region	88.93	<0.0001	****	Yes
Age	0.32	0.3940	ns	No
Treatment	0.59	0.2565	ns	No
Region x Age	0.20	0.7617	ns	No
Region x Treatment	1.54	0.0155	*	Yes
Age x Treatment	1.48	0.0899	ns	No
Region x Age x Treatment	0.34	0.5338	ns	No

****P<0.0001, ***P<0.001, **P<0.01, *P<0.05, ns not significant

Table S2. Multiple comparisons of the PC1 score values among different groups and brain regions, with Tukey's correction.

Tukey's multiple comparisons test	Mean Diff.	95.00% CI of diff.	Summary	Adj. P Value
CBL:14-m C vs. CBL:14-m T	-5.899	-34.63 to 22.84	ns	0.914
CBL:14-m C vs. CBL:12-w C	3.337	-45.58 to 52.26	ns	>0.9999
CBL:14-m C vs. CBL:12-w T	3.763	-11.42 to 18.95	ns	0.784
CBL:14-m C vs. CPu:14-m C	0.3138	-30.97 to 31.59	ns	>0.9999
CBL:14-m C vs. CPu:14-m T	-5.329	-68.95 to 58.29	ns	0.999
CBL:14-m C vs. CPu:12-w C	-2.362	-62.97 to 58.24	ns	>0.9999
CBL:14-m C vs. CPu:12-w T	11.41	-2.752 to 25.58	ns	0.095
CBL:14-m C vs. CTX:14-m C	12.51	-9.734 to 34.75	ns	0.148
CBL:14-m C vs. CTX:14-m T	2.417	-71.71 to 76.55	ns	>0.9999
CBL:14-m C vs. CTX:12-w C	2.064	-142.4 to 146.5	ns	>0.9999
CBL:14-m C vs. CTX:12-w T	15.67	1.002 to 30.33	*	0.042
CBL:14-m C vs. HB:14-m C	-74.72	-227.4 to 77.96	ns	0.190
CBL:14-m C vs. HB:14-m T	-58.55	-115.7 to -1.413	*	0.047
CBL:14-m C vs. HB:12-w C	-75.62	-161.5 to 10.27	ns	0.065
CBL:14-m C vs. HB:12-w T	-52.93	-68.94 to -36.92	**	0.004
CBL:14-m C vs. HC:14-m C	18.98	-2.827 to 40.78	ns	0.065
CBL:14-m C vs. HC:14-m T	10.67	-37.26 to 58.60	ns	0.771
CBL:14-m C vs. HC:12-w C	9.632	-63.99 to 83.25	ns	0.958
CBL:14-m C vs. HC:12-w T	20.54	1.465 to 39.61	*	0.039
CBL:14-m T vs. CBL:12-w C	9.237	-32.62 to 51.09	ns	0.933
CBL:14-m T vs. CBL:12-w T	9.663	-24.81 to 44.13	ns	0.536
CBL:14-m T vs. CPu:14-m C	6.213	-26.39 to 38.82	ns	0.848

CBL:14-m T vs. CPu:14-m T	0.5702	-39.09 to 40.23	ns	>0.9999
CBL:14-m T vs. CPu:12-w C	3.538	-47.75 to 54.83	ns	>0.9999
CBL:14-m T vs. CPu:12-w T	17.31	-14.87 to 49.49	ns	0.196
CBL:14-m T vs. CTX:14-m C	18.41	-18.62 to 55.44	ns	0.191
CBL:14-m T vs. CTX:14-m T	8.317	-44.68 to 61.32	ns	0.838
CBL:14-m T vs. CTX:12-w C	7.964	-126.0 to 141.9	ns	>0.9999
CBL:14-m T vs. CTX:12-w T	21.57	-12.06 to 55.19	ns	0.130
CBL:14-m T vs. HB:14-m C	-68.82	-195.5 to 57.82	ns	0.180
CBL:14-m T vs. HB:14-m T	-52.65	-90.50 to -14.79	*	0.026
CBL:14-m T vs. HB:12-w C	-69.72	-144.4 to 4.939	ns	0.059
CBL:14-m T vs. HB:12-w T	-47.03	-82.48 to -11.58	*	0.028
CBL:14-m T vs. HC:14-m C	24.88	-12.10 to 61.85	ns	0.109
CBL:14-m T vs. HC:14-m T	16.57	-9.151 to 42.29	ns	0.116
CBL:14-m T vs. HC:12-w C	15.53	-47.42 to 78.48	ns	0.788
CBL:14-m T vs. HC:12-w T	26.44	-0.9495 to 53.82	ns	0.056
CBL:12-w C vs. CBL:12-w T	0.4258	-54.85 to 55.71	ns	>0.9999
CBL:12-w C vs. CPu:14-m C	-3.024	-56.63 to 50.58	ns	>0.9999
CBL:12-w C vs. CPu:14-m T	-8.667	-60.82 to 43.48	ns	0.995
CBL:12-w C vs. CPu:12-w C	-5.699	-54.07 to 42.67	ns	0.949
CBL:12-w C vs. CPu:12-w T	8.077	-45.11 to 61.27	ns	0.906
CBL:12-w C vs. CTX:14-m C	9.172	-48.08 to 66.42	ns	0.835
CBL:12-w C vs. CTX:14-m T	-0.9199	-59.76 to 57.92	ns	>0.9999
CBL:12-w C vs. CTX:12-w C	-1.273	-169.2 to 166.7	ns	>0.9999
CBL:12-w C vs. CTX:12-w T	12.33	-42.22 to 66.87	ns	0.657
CBL:12-w C vs. HB:14-m C	-78.05	-192.7 to 36.56	ns	0.126
CBL:12-w C vs. HB:14-m T	-61.88	-110.5 to -13.23	*	0.022
CBL:12-w C vs. HB:12-w C	-78.95	-224.5 to 66.59	ns	0.158
CBL:12-w C vs. HB:12-w T	-56.27	-112.3 to -0.1895	*	0.050
CBL:12-w C vs. HC:14-m C	15.64	-41.58 to 72.85	ns	0.489
CBL:12-w C vs. HC:14-m T	7.332	-37.40 to 52.06	ns	0.996
CBL:12-w C vs. HC:12-w C	6.294	-53.16 to 65.75	ns	0.971
CBL:12-w C vs. HC:12-w T	17.2	-27.94 to 62.34	ns	0.443
CBL:12-w T vs. CPu:14-m C	-3.449	-12.17 to 5.269	ns	0.559
CBL:12-w T vs. CPu:14-m T	-9.092	-78.73 to 60.55	ns	0.932
CBL:12-w T vs. CPu:12-w C	-6.125	-72.83 to 60.58	ns	0.991
CBL:12-w T vs. CPu:12-w T	7.652	-0.2035 to 15.51	ns	0.053
CBL:12-w T vs. CTX:14-m C	8.746	1.762 to 15.73	*	0.027
CBL:12-w T vs. CTX:14-m T	-1.346	-81.16 to 78.47	ns	>0.9999
CBL:12-w T vs. CTX:12-w C	-1.699	-150.0 to 146.6	ns	>0.9999
CBL:12-w T vs. CTX:12-w T	11.9	-6.445 to 30.25	ns	0.114
CBL:12-w T vs. HB:14-m C	-78.48	-219.8 to 62.81	ns	0.153
CBL:12-w T vs. HB:14-m T	-62.31	-125.6 to 1.019	ns	0.052
CBL:12-w T vs. HB:12-w C	-79.38	-170.6 to 11.82	ns	0.066
CBL:12-w T vs. HB:12-w T	-56.69	-66.65 to -46.74	****	<0.0001

CBL:12-w T vs. HC:14-m C	15.21	8.274 to 22.15	**	0.005
CBL:12-w T vs. HC:14-m T	6.906	-47.40 to 61.21	ns	0.947
CBL:12-w T vs. HC:12-w C	5.868	-73.46 to 85.19	ns	0.998
CBL:12-w T vs. HC:12-w T	16.77	-14.99 to 48.53	ns	0.166
CPu:14-m C vs. CPu:14-m T	-5.643	-73.79 to 62.51	ns	0.997
CPu:14-m C vs. CPu:12-w C	-2.675	-67.86 to 62.51	ns	>0.9999
CPu:14-m C vs. CPu:12-w T	11.1	1.399 to 20.80	*	0.031
CPu:14-m C vs. CTX:14-m C	12.2	0.1680 to 24.22	*	0.049
CPu:14-m C vs. CTX:14-m T	2.104	-76.35 to 80.56	ns	>0.9999
CPu:14-m C vs. CTX:12-w C	1.75	-145.7 to 149.2	ns	>0.9999
CPu:14-m C vs. CTX:12-w T	15.35	6.438 to 24.27	**	0.007
CPu:14-m C vs. HB:14-m C	-75.03	-216.2 to 66.10	ns	0.164
CPu:14-m C vs. HB:14-m T	-58.86	-120.6 to 2.898	ns	0.055
CPu:14-m C vs. HB:12-w C	-75.93	-165.9 to 14.03	ns	0.070
CPu:14-m C vs. HB:12-w T	-53.24	-62.04 to -44.45	***	0.000
CPu:14-m C vs. HC:14-m C	18.66	5.981 to 31.34	*	0.022
CPu:14-m C vs. HC:14-m T	10.36	-42.26 to 62.97	ns	0.767
CPu:14-m C vs. HC:12-w C	9.318	-68.64 to 87.27	ns	0.959
CPu:14-m C vs. HC:12-w T	20.22	-0.4283 to 40.87	ns	0.053
CPu:14-m T vs. CPu:12-w C	2.968	-51.90 to 57.84	ns	>0.9999
CPu:14-m T vs. CPu:12-w T	16.74	-51.03 to 84.52	ns	0.593
CPu:14-m T vs. CTX:14-m C	17.84	-53.46 to 89.14	ns	0.545
CPu:14-m T vs. CTX:14-m T	7.747	-7.090 to 22.58	ns	0.170
CPu:14-m T vs. CTX:12-w C	7.393	-107.4 to 122.2	ns	>0.9999
CPu:14-m T vs. CTX:12-w T	21	-48.00 to 89.99	ns	0.438
CPu:14-m T vs. HB:14-m C	-69.39	-177.5 to 38.76	ns	0.158
CPu:14-m T vs. HB:14-m T	-53.22	-63.67 to -42.77	****	<0.0001
CPu:14-m T vs. HB:12-w C	-70.29	-137.4 to -3.182	*	0.043
CPu:14-m T vs. HB:12-w T	-47.6	-117.9 to 22.72	ns	0.107
CPu:14-m T vs. HC:14-m C	24.31	-46.97 to 95.58	ns	0.352
CPu:14-m T vs. HC:14-m T	16	1.063 to 30.93	*	0.044
CPu:14-m T vs. HC:12-w C	14.96	-45.28 to 75.20	ns	0.925
CPu:14-m T vs. HC:12-w T	25.87	-33.49 to 85.22	ns	0.309
CPu:12-w C vs. CPu:12-w T	13.78	-51.02 to 78.57	ns	0.702
CPu:12-w C vs. CTX:14-m C	14.87	-53.55 to 83.30	ns	0.642
CPu:12-w C vs. CTX:14-m T	4.779	-55.09 to 64.65	ns	>0.9999
CPu:12-w C vs. CTX:12-w C	4.426	-118.7 to 127.6	ns	>0.9999
CPu:12-w C vs. CTX:12-w T	18.03	-48.02 to 84.07	ns	0.512
CPu:12-w C vs. HB:14-m C	-72.36	-181.6 to 36.93	ns	0.144
CPu:12-w C vs. HB:14-m T	-56.18	-108.7 to -3.676	*	0.040
CPu:12-w C vs. HB:12-w C	-73.25	-202.4 to 55.86	ns	0.146
CPu:12-w C vs. HB:12-w T	-50.57	-118.0 to 16.84	ns	0.088
CPu:12-w C vs. HC:14-m C	21.34	-47.06 to 89.73	ns	0.403
CPu:12-w C vs. HC:14-m T	13.03	-37.26 to 63.32	ns	0.901

CPu:12-w C vs. HC:12-w C	11.99	-1.011 to 25.00	ns	0.058
CPu:12-w C vs. HC:12-w T	22.9	-33.49 to 79.29	ns	0.359
CPu:12-w T vs. CTX:14-m C	1.095	-9.597 to 11.79	ns	0.997
CPu:12-w T vs. CTX:14-m T	-8.997	-87.10 to 69.11	ns	0.968
CPu:12-w T vs. CTX:12-w C	-9.351	-156.6 to 137.9	ns	1.000
CPu:12-w T vs. CTX:12-w T	4.252	-13.73 to 22.24	ns	0.577
CPu:12-w T vs. HB:14-m C	-86.13	-226.3 to 54.07	ns	0.128
CPu:12-w T vs. HB:14-m T	-69.96	-131.3 to -8.598	*	0.038
CPu:12-w T vs. HB:12-w C	-87.03	-176.7 to 2.603	ns	0.053
CPu:12-w T vs. HB:12-w T	-64.34	-81.27 to -47.42	**	0.002
CPu:12-w T vs. HC:14-m C	7.561	-3.061 to 18.18	ns	0.110
CPu:12-w T vs. HC:14-m T	-0.7453	-52.94 to 51.45	ns	>0.9999
CPu:12-w T vs. HC:12-w C	-1.783	-79.39 to 75.82	ns	>0.9999
CPu:12-w T vs. HC:12-w T	9.122	-18.20 to 36.44	ns	0.360
CTX:14-m C vs. CTX:14-m T	-10.09	-91.40 to 71.21	ns	0.937
CTX:14-m C vs. CTX:12-w C	-10.45	-159.6 to 138.7	ns	0.999
CTX:14-m C vs. CTX:12-w T	3.157	-5.160 to 11.47	ns	0.429
CTX:14-m C vs. HB:14-m C	-87.23	-225.2 to 50.76	ns	0.120
CTX:14-m C vs. HB:14-m T	-71.05	-136.2 to -5.941	*	0.043
CTX:14-m C vs. HB:12-w C	-88.13	-180.6 to 4.398	ns	0.055
CTX:14-m C vs. HB:12-w T	-65.44	-70.95 to -59.93	****	<0.0001
CTX:14-m C vs. HC:14-m C	6.466	5.812 to 7.121	****	<0.0001
CTX:14-m C vs. HC:14-m T	-1.84	-58.14 to 54.46	ns	>0.9999
CTX:14-m C vs. HC:12-w C	-2.878	-83.70 to 77.94	ns	>0.9999
CTX:14-m C vs. HC:12-w T	8.027	-17.09 to 33.14	ns	0.415
CTX:14-m T vs. CTX:12-w C	-0.3533	-111.5 to 110.8	ns	>0.9999
CTX:14-m T vs. CTX:12-w T	13.25	-65.98 to 92.48	ns	0.826
CTX:14-m T vs. HB:14-m C	-77.13	-182.1 to 27.86	ns	0.118
CTX:14-m T vs. HB:14-m T	-60.96	-77.11 to -44.82	**	0.002
CTX:14-m T vs. HB:12-w C	-78.03	-146.8 to -9.254	*	0.032
CTX:14-m T vs. HB:12-w T	-55.35	-135.8 to 25.09	ns	0.103
CTX:14-m T vs. HC:14-m C	16.56	-64.72 to 97.84	ns	0.684
CTX:14-m T vs. HC:14-m T	8.252	-19.05 to 35.55	ns	0.417
CTX:14-m T vs. HC:12-w C	7.214	-56.25 to 70.68	ns	>0.9999
CTX:14-m T vs. HC:12-w T	18.12	-51.66 to 87.90	ns	0.647
CTX:12-w C vs. CTX:12-w T	13.6	-134.3 to 161.5	ns	0.989
CTX:12-w C vs. HB:14-m C	-76.78	-190.5 to 36.94	ns	0.175
CTX:12-w C vs. HB:14-m T	-60.61	-178.2 to 57.00	ns	0.242
CTX:12-w C vs. HB:12-w C	-77.68	-192.2 to 36.87	ns	0.105
CTX:12-w C vs. HB:12-w T	-54.99	-203.6 to 93.62	ns	0.308
CTX:12-w C vs. HC:14-m C	16.91	-132.2 to 166.0	ns	0.958
CTX:12-w C vs. HC:14-m T	8.605	-113.7 to 130.9	ns	>0.9999
CTX:12-w C vs. HC:12-w C	7.567	-109.9 to 125.0	ns	0.999
CTX:12-w C vs. HC:12-w T	18.47	-122.4 to 159.4	ns	0.942

CTX:12-w T vs. HB:14-m C	-90.38	-231.3 to 50.54	ns	0.118
CTX:12-w T vs. HB:14-m T	-74.21	-136.9 to -11.57	*	0.036
CTX:12-w T vs. HB:12-w C	-91.28	-181.9 to -0.6177	*	0.049
CTX:12-w T vs. HB:12-w T	-68.6	-85.98 to -51.21	**	0.001
CTX:12-w T vs. HC:14-m C	3.309	-4.950 to 11.57	ns	0.397
CTX:12-w T vs. HC:14-m T	-4.997	-58.56 to 48.57	ns	0.993
CTX:12-w T vs. HC:12-w C	-6.035	-84.77 to 72.70	ns	0.998
CTX:12-w T vs. HC:12-w T	4.87	-12.24 to 21.98	ns	0.455
HB:14-m C vs. HB:14-m T	16.17	-94.55 to 126.9	ns	0.983
HB:14-m C vs. HB:12-w C	-0.898	-103.9 to 102.1	ns	>0.9999
HB:14-m C vs. HB:12-w T	21.79	-119.9 to 163.5	ns	0.852
HB:14-m C vs. HC:14-m C	93.69	-44.19 to 231.6	ns	0.105
HB:14-m C vs. HC:14-m T	85.39	-29.74 to 200.5	ns	0.103
HB:14-m C vs. HC:12-w C	84.35	-20.77 to 189.5	ns	0.093
HB:14-m C vs. HC:12-w T	95.25	-38.45 to 229.0	ns	0.100
HB:14-m T vs. HB:12-w C	-17.07	-83.94 to 49.80	ns	0.883
HB:14-m T vs. HB:12-w T	5.616	-58.44 to 69.67	ns	0.993
HB:14-m T vs. HC:14-m C	77.52	12.44 to 142.6	*	0.036
HB:14-m T vs. HC:14-m T	69.21	56.70 to 81.73	****	<0.0001
HB:14-m T vs. HC:12-w C	68.18	9.142 to 127.2	*	0.031
HB:14-m T vs. HC:12-w T	79.08	26.08 to 132.1	*	0.019
HB:12-w C vs. HB:12-w T	22.69	-69.06 to 114.4	ns	0.557
HB:12-w C vs. HC:14-m C	94.59	2.091 to 187.1	*	0.048
HB:12-w C vs. HC:14-m T	86.29	18.56 to 154.0	*	0.024
HB:12-w C vs. HC:12-w C	85.25	-49.95 to 220.4	ns	0.120
HB:12-w C vs. HC:12-w T	96.15	14.58 to 177.7	*	0.035
HB:12-w T vs. HC:14-m C	71.91	66.42 to 77.39	****	<0.0001
HB:12-w T vs. HC:14-m T	63.6	8.487 to 118.7	*	0.038
HB:12-w T vs. HC:12-w C	62.56	-17.38 to 142.5	ns	0.081
HB:12-w T vs. HC:12-w T	73.47	39.53 to 107.4	*	0.013
HC:14-m C vs. HC:14-m T	-8.306	-64.57 to 47.96	ns	0.876
HC:14-m C vs. HC:12-w C	-9.344	-90.14 to 71.45	ns	0.955
HC:14-m C vs. HC:12-w T	1.561	-23.49 to 26.61	ns	1.000
HC:14-m T vs. HC:12-w C	-1.038	-59.55 to 57.47	ns	>0.9999
HC:14-m T vs. HC:12-w T	9.867	-34.34 to 54.08	ns	0.848
HC:12-w C vs. HC:12-w T	10.9	-58.37 to 80.18	ns	0.933

****P<0.0001, ***P<0.001, **P<0.01, *P<0.05, ns not significant

Table S3. List of significantly altered features based on limma analysis.

Metabolite Assignment	Ion Type	Molecular Formula	m/z theor.	m/z exper.	ppm error	Identification	P[a]	logFC[b]
Sulfoacetic acid	M-H	$C_2H_4O_5S$	138.9707	138.9708	-0.36	Mass accuracy[1]	1.4E-05	0.60
Glutamine	M-H	$C_5H_{10}N_2O_3$	145.0619	145.0619	-0.28	MS/MS	3.2E-02	0.49
				154.9188			6.1E-06	0.66
				170.8927			6.8E-04	0.60
				172.8320			4.8E-02	0.55
				198.9085			3.8E-05	0.63
				201.4858			5.3E-07	0.64
				201.6435			6.3E-04	-0.50
		$C_5H_8O_7S$	210.9918	210.9920	-0.90	*SM	3.6E-06	0.60
		$C_5H_{10}O_7S$	213.0075	213.0077	-0.94	*SM	1.1E-06	0.61
				222.9200			3.1E-02	-0.23
		$C_6H_{10}O_7S$	225.0075	225.0076	-0.76	*SM	4.5E-07	0.68
Carnosine	M-H	$C_9H_{14}N_4O_3$	225.0993	225.0996	-1.38	MS/MS[2]	4.0E-11	1.11
		$C_{13}H_{25}NS$	226.1635	226.1638	-1.55	*SM	1.2E-15	1.38
				226.4150			3.0E-15	1.31
				226.6684			1.4E-07	0.71
		$C_6H_8O_8S$	238.9867	238.9869	-0.63	*SM	6.7E-06	0.60
Product ion of SHex-Cer/loss of water from the sulfated hexose head group	M-H2O-H	$C_6H_9O_8S$	241.0024	241.0025	-0.33		1.4E-06	0.58
				242.9982			2.3E-07	0.64
		$C_6H_{12}O_8S$	243.0180	243.0182	-0.66	*SM	6.8E-08	0.76
		$C_6H_{10}O_9S$	256.9973	256.9973	-0.23	*SM	2.1E-06	0.58
Product ion of SHex-Cer/ sulfated hexose head group	M-H	$C_6H_{12}O_9S$	259.0129	259.0130	-0.42		1.3E-07	0.64
				261.0087			6.0E-08	0.73
		$C_9H_{16}O_9S$	299.0442	299.0442	0.20	*SM	3.9E-16	-1.09
N-Acetylgalacto-samine 6-sulfate	M-H	$C_8H_{15}NO_9S$	300.0395	300.0393	0.60	*SM	9.2E-07	0.80
				302.0354			2.9E-08	0.79
		$C_8H_{17}NO_9S$	302.0551	302.0551	-0.03	*SM	6.4E-11	1.07
		$C_9H_{15}NO_{10}S$	328.0344	328.0342	0.49	*SM	2.6E-09	0.88
		$C_{10}H_{17}NO_{10}S$	342.0500	342.0500	0.03	*SM	9.0E-06	0.60
				357.4599			3.2E-09	1.35
Product ion of SHex-Cer		$C_{26}H_{50}NO_{10}S$	568.3161	568.3157	0.70	Mass accuracy[3]	2.8E-11	-1.18
		$C_{26}H_{49}NO_{11}S$	582.2954	582.2948	1.00	*SM	1.4E-07	-0.96
SHexCer(d36:1)	M-H	$C_{42}H_{81}NO_{11}S$	806.5458	806.5444	1.67	DESI MRM	8.2E-17	-1.56
SHexCer(t18:1/18:0)	M-H	$C_{42}H_{81}NO_{12}S$	822.5407	822.5386	2.52	DESI MRM	1.2E-08	-1.28
SHexCer 40:3;O2	M-H	$C_{46}H_{85}NO_{11}S$	858.5771	858.5734	4.31	Mass accuracy[4]	2.1E-03	0.71

SHexCer(t39:1)	M-H	$C_{45}H_{87}NO_{12}S$	864.5876	864.5862	1.58	Mass accuracy[5]	3.0E-15	1.62
C24:1 Sulf. -H2O			870.6134	870.6122	1.38	Mass accuracy[6]	1.5E-03	0.72
SHexCer(d41:2)	M-H	$C_{47}H_{89}NO_{11}S$	874.6084	874.6045	4.46	DESI MRM	7.6E-12	1.38
SHexCer(d18:1/24:1)	M-H	$C_{48}H_{91}NO_{11}S$	888.6240	888.6246	-0.71	DESI MRM	2.4E-06	0.68
SHexCer(d18:1:h23:0)	M-H	$C_{47}H_{91}NO_{12}S$	892.6189	892.6179	1.17	Mass accuracy[5]	2.4E-17	1.58
SHex-Cer(d18:1:h23:0)_3rd isotope	M-H	$C_{47}H_{91}NO_{12}S$	894.6230	894.6224	0.68	Mass accuracy[5]	6.7E-03	0.80
		$C_{49}H_{91}NO_{11}S$	900.6240	900.6204	4.00	Mass accuracy[4]	1.1E-09	1.42
SHexCer(d18:1/25:1)	M-H	$C_{49}H_{93}NO_{11}S$	902.6397	902.6364	3.59	Mass accuracy[5]	3.2E-04	1.00
SHex-Cer(d18:1/h24:1)	M-H	$C_{48}H_{91}NO_{12}S$	904.6189	904.6177	1.35	DESI MRM	9.9E-11	1.20
SHex-Cer(d18:1/h25:1)	M-H	$C_{49}H_{93}NO_{12}S$	918.6346	918.6363	-1.85	Mass accuracy[5]	3.4E-06	1.16
SHex-Cer(d18:1/h25:0)_2nd isotope	M-H	$C_{49}H_{95}NO_{12}S$	921.6535	921.6518	1.92	Mass accuracy[5]	5.0E-03	0.79
SHex-Cer(d18:1/h26:1)_2nd isotope	M-H	$C_{50}H_{95}NO_{12}S$	933.6535	933.6506	3.19	Mass accuracy[5]	2.5E-02	0.56

*SM, smart formula (molecular formula prediction tool-based result from the m/z value and expected atomic composition)

[a] Corrected P value from a limma analysis which compares the two different age groups (12-w and 14-m) with treatment (tacrine/control) and different brain regions (CB, CPu, CTX, HB, HIP) serving as co-variates.

[b]$\log_2$ of the fold change (FC) 14-m/12-w (i.e., positive values indicate increase with age).

Table S4. List of features from the ROC analysis and their corresponding AUC values.

m/z	AUC	*m/z*	AUC	*m/z*	AUC
**902.640**	0.000	**701.513**	0.170	**729.542**	0.863
**874.609**	0.000	**832.608**	0.177	**757.531**	0.879
**892.617**	0.001	**888.623**	0.179	**793.057**	0.894
**892.623**	0.001	**808.592**	0.183	**864.571**	0.899
**893.623**	0.001	**848.595**	0.184	**892.643**	0.902
**904.621**	0.001	**848.595**	0.184	**820.498**	0.905
**903.643**	0.001	**788.545**	0.202	**918.668**	0.915
**905.622**	0.002	**788.539**	0.204	**865.575**	0.917
**894.625**	0.003	**788.544**	0.205	**805.995**	0.924
**918.636**	0.003	**664.375**	0.205	**837.545**	0.924
**902.641**	0.005	**703.521**	0.216	**822.539**	0.932
**876.623**	0.012	**946.584**	0.221	**822.539**	0.932
**902.637**	0.024	**876.589**	0.232	**820.525**	0.947
**902.637**	0.024	**962.493**	0.233	**862.609**	0.949
**848.640**	0.025	**846.491**	0.238	**780.520**	0.953
**900.625**	0.050	**902.609**	0.238	**864.615**	0.983
**920.650**	0.053	**903.607**	0.242	**890.638**	0.983
**904.618**	0.055	**902.605**	0.246	**865.624**	0.986
**806.591**	0.063	**822.579**	0.247	**781.479**	0.988
**865.593**	0.092	**852.584**	0.750	**795.539**	0.990
**876.620**	0.095	**930.631**	0.760	**864.618**	0.993
**901.628**	0.099	**502.290**	0.762	**822.542**	0.997
**850.640**	0.103	**865.546**	0.768	**804.529**	0.998
**921.656**	0.104	**917.631**	0.770	**809.556**	0.999
**806.593**	0.109	**934.665**	0.776	**821.529**	0.999
**919.624**	0.115	**834.575**	0.776	**809.516**	1.000
**652.375**	0.123	**508.340**	0.777	**806.545**	1.000
**652.375**	0.123	**862.605**	0.778	**807.550**	1.000
**807.596**	0.133	**836.538**	0.780	**822.542**	1.000
**904.810**	0.137	**881.614**	0.791		
**812.663**	0.139	**796.560**	0.795		
**1572.904**	0.143	**758.577**	0.803		
**860.638**	0.150	**784.507**	0.808		
**860.639**	0.150	**539.290**	0.819		
**860.640**	0.150	**914.641**	0.821		
**810.527**	0.154	**816.555**	0.842		
**810.529**	0.154	**772.587**	0.843		
**788.545**	0.154	**337.311**	0.845		
**789.551**	0.156	**844.605**	0.846		
**864.587**	0.166	**916.660**	0.862		

References

(1) Wishart, D. S., et al., HMDB: the Human Metabolome Database. *Nucleic Acids Res* 2007, **35** (Database issue), D521-6.

(2) Vallianatou, T., et al. Integration of mass spectrometry imaging and machine learning visualizes region-specific age-induced and drug-target metabolic perturbations in the brain. *ACS Chem Neurosci* 2021, **12** (10), 1811-1823.

(3) Hsu, F. F.; Turk, J. Studies on sulfatides by quadrupole ion-trap mass spectrometry with electrospray ionization: structural characterization and the fragmentation processes that include an unusual internal galactose residue loss and the classical charge-remote fragmentation. *J Am Soc Mass Spectrom* 2004, **15** (4), 536-46.

(4) O'Donnell, V. B., et al. LIPID MAPS: Serving the next generation of lipid researchers with tools, resources, data, and training. *Sci Signal* 2019, **12** (563).

(5) Kaya, I., et al. Spatial lipidomics reveals brain region-specific changes of sulfatides in an experimental MPTP Parkinson's disease primate model. *NPJ Parkinsons Dis* 2023, **9** (1), 118.

(6) Angerer, T. B., et al. Evaluation of 6 MALDI-matrices for 10 µm lipid imaging and on-tissue MSn with AP-MALDI-Orbitrap. *J Am Soc Mass Spectrom* 2022, **33** (5), 760-771.
